# Carbon‐Coated Urchin‐Like Silica Nanospheres for Enhanced Photothermal Catalysis

**DOI:** 10.1002/cssc.202500068

**Published:** 2025-04-08

**Authors:** Alejandra Rendon‐Patiño, Xinhuilan Wang, Stiven Duran‐Uribe, Antonio Sepulveda‐Escribano, Diego Mateo, Enrique V. Ramos‐Fernandez, Jorge Gascon

**Affiliations:** ^1^ KAUST Catalysis Center King Abdullah University of Science and Technology Thuwal 23955‐6900 Saudi Arabia; ^2^ Laboratory of Advanced Materials Inorganic Chemistry Department University Materials Institute of Alicante University of Alicante Apartado 99 E‐03080 Alicante Spain

**Keywords:** ammonia decomposition, CO_2_ hydrogenation, photothermal catalysis, ruthenium nanoparticles

## Abstract

Photothermal catalysis represents a promising strategy to improve the sustainability of chemical transformations by integrating light and heat into a single process. However, materials featuring excellent harvesting and utilization of solar energy are still needed. Here, a photothermal catalyst architecture is reported, which is based on carbon‐coated urchin‐like silica nanospheres (KCC‐1) decorated with Ru nanoparticles that maximizes light absorption and heat confinement. The composite material exhibits outstanding catalytic activity toward photothermal ammonia decomposition and CO_2_ hydrogenation reactions, outperforming most traditional Ru‐based thermal catalysts. The insulating nature of silica is hypothesized to help minimize heat loss via conduction, while its high surface area enables excellent metal dispersion. Additionally, the deposition of a carbon layer further enhances both photon absorption and light‐to‐heat conversion. Mechanistic experiments suggest the co‐existence of thermal and nonthermal effects, with light playing a crucial role in facilitating the desorption of H_2_ and N_2_ from the surface of the catalyst. Overall, these results demonstrate that the rational design of catalysts combining effective heat insulators and broad light absorbers is crucial to optimizing catalytic performance in photothermal systems.

## Graphical abstract

An advanced catalytic architecture based on carbon‐coated, urchin‐like silica nanospheres decorated with Ru nanoparticles is presented. This composite optimizes light absorption and heat retention, significantly enhancing NH_3_ decomposition and CO_2_ hydrogenation via synergistic thermal and photoinduced effects, as evidenced by photocurrent and time‐resolved photoluminescence measurements.

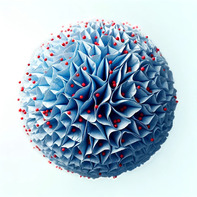

## 1 Introduction

Through the synergistic combination of photo‐ and thermochemical contributions of sunlight, photothermal catalysis has the potential to enhance reaction rates and to change selectivity patterns, even at moderate operation conditions. Photothermal catalysis allows for a more effective harvesting of the solar spectrum, including low‐energy visible and infrared photons that would be insufficient to promote photocatalytic reactions. Furthermore, thanks to the increase in the temperature of the catalytic active sites, photothermal catalysis renders outstanding production rates while operating at moderate conditions. A photothermal catalyst should, therefore, be able to absorb light and convert it into heat while activating adsorbates *via* vibrational or electronic transitions. Facilitating in this way chemical reactions at lower temperatures than would be required by conventional thermal catalysis. These catalysts typically consist of metals, metal oxides, or composite structures endowed with specific optical and thermal properties to maximize light absorption and thermal conversion.^[^ ^1^, ^2^, ^3^, ^4^, ^5^ ^]^

In photothermal catalysis, it is crucial to maximize sunlight absorption and minimize the dissipation of thermal energy to enhance the efficiency of sunlight utilization. Additionally, reducing light reflection is essential to ensure optimal energy capture. During this process, heat can be lost through conduction, convection, and radiation, largely due to the significant temperature difference between the catalyst and its surroundings.^[^ ^6^, ^7^, ^8^, ^9^, ^10^, ^11^ ^]^ As a result, a substantial amount of the thermal energy converted from sunlight is lost to the environment. Consequently, reducing heat loss in catalysts has become a crucial challenge in the field.

To reduce light reflection, photothermal materials can be fabricated as highly porous nanostructures at the meso‐macroscopic level. These structures have a very low‐ effective refractive index, which significantly reduces light reflection.^[^ ^12^, ^13^, ^14^, ^15^ ^]^ From a microscopic point of view, however, the porous configuration causes light to undergo multiple reflections within the pores, thus increasing the probability for photon absorption.^[^ ^15^ ^]^ For instance, hierarchical graphene foam^[^ ^16^ ^]^ and cauliflower‐shaped hierarchical copper nanostructures^[^ ^15^ ^]^ have all demonstrated significantly lower sensitivity to the incident angle of sunlight, thereby improving their efficiency in solar energy applications.^[^ ^17^ ^]^

Numerous efforts have been undertaken to decrease heat conduction in nanomaterials. For example, metal nanoparticles, that efficiently convert light into heat, have been coated with insulating layers to simultaneously enhance light absorption and reduce heat conduction.^[^ ^14^, ^18^, ^19^, ^20^, ^21^, ^22^, ^23^, ^24^, ^25^, ^26^ ^]^ The nanostructured heat insulation approaches mentioned previously are highly effective at blocking thermal conduction but not as effective against thermal radiation.^[^ ^27^, ^28^ ^]^ Thermal radiation fundamentally consists of infrared (IR) light, predominantly spanning wavelengths of 4–100 μm, characterized by its strong penetration ability that allows it to pass through many substances or even vacuum. For this reason, to enhance the retention of sunlight‐converted thermal energy, an additional strategy is required to reduce the thermal radiation of photothermal materials.^[^ ^21^ ^]^

Therefore, from the perspective of material design, a photothermal catalyst with complex porosity is essential, allowing light to penetrate and reflect within its voids, thus concentrating the light internally. Additionally, metal nanoparticles located within the porous structure should be employed to convert light into heat and facilitate chemical transformations. Last but not least, the catalyst should exhibit poor thermal conductivity and minimized thermal emission to improve heat confinement.

Taking into account all these premises, in this study we have developed a composite material with improved photothermal properties. In particular, we selected urchin‐like silica nanospheres KCC‐1 (where KCC stands for “KAUST Catalysis Center”) as a mesoporous silica core featuring an intricate porosity and a low thermal conductivity that are advantageous to prevent light reflection and hinder heat conduction to the exterior.^[^ ^20^, ^27^, ^28^ ^]^ Subsequently, the surface of KCC‐1 has been decorated with catalytic Ru sites coated with a thin carbon layer to enhance light absorption and heat generation within the structure. In addition, the urchin‐like silica traps infrared light radiated from the carbon shell, thus minimizing the heat loss through radiation.^[^ ^29^, ^30^ ^]^ The as‐prepared composite has been tested in both the photothermal NH_3_ decomposition and CO_2_ hydrogenation reactions, displaying excellent catalytic performances that outperform the current state‐of‐the‐art for conventional thermal catalysts. Mechanistic studies suggest that the remarkable catalytic activities stem from the synergistic combination of thermal and nonthermal effects, particularly at high irradiances. Overall, these results demonstrate that the rational design of catalysts combining effective heat insulators and broad light absorbers is crucial to maximize the catalytic performance in photothermal systems.

## 2 Results and Discussion

First of all, and for comparison purposes, spherical silica particles were synthesized using the Stöber method. On the contrary, KCC‐1 was obtained following the previously reported protocol.^[^ ^31^, ^32^ ^]^ Figure S1, Supporting Information, displays scanning electron mircroscopy images of both materials with the expected morphology.

Literature suggests that surface functionalization of SiO_2_ with amino groups improves the dispersion of ruthenium.^[^ ^33^, ^34^, ^35^ ^]^ Therefore, both silica spheres (SiO_2_) and KCC‐1 were functionalized using aminopropyltriethoxysilane (APTS) prior to the introduction of ruthenium (see experimental details in the Supporting Information). Infrared spectroscopy was employed to demonstrate the successful functionalization of both materials.^[^ ^36^, ^37^ ^]^ As can be seen in the infrared spectra, upon amino functionalization the vibrations of the —OH groups decrease in intensity (Figure S2, Supporting Information). Furthermore, new peaks appear between 2900 and 3000 cm^−1^ and at 1580 cm^−1^ corresponding to the C—H vibration frequencies and —NH_2_ stretching from the APTS functionalization. The main difference between both materials is the intensity of these peaks. In the KCC‐1 sample, peaks are more intense, indicating a higher degree of functionalization. In fact, thermogravimetric data further confirmed these observations owing to the greater amount of organic matter present in the aminated KCC‐1 sample (KCC‐1‐NH_2_) compared to the aminated silica spheres (SiO_2_‐NH_2_) (Figure S3 and S4, Supporting Information). These results are related to the number of —OH groups present on the surface, which also depend on the surface area of both materials. In fact, the calculated surface area of KCC‐1 was 445 m^2^ g^−1^,^[^ ^31^ ^]^ while spherical SiO_2_ displayed a value of 48 m^2^ g^−1^ (Figure S5, Supporting Information).

In the subsequent stage, the materials were impregnated with RuCl_3_·*x*H_2_O using the incipient wetness impregnation method. Subsequently, samples were annealed under N_2_ atmosphere at 600 °C to decompose the RuCl_3_·*x*H_2_O. The metal loading for both samples was determined by TGA and ICP, achieving 1.55 ± 0.18 and 1.45 ± 0.15 wt% Ru for SiO_2_‐NH_2_‐Ru and KCC‐1‐NH_2_‐Ru, respectively (Figure S4 and Table S1 and S2, Supporting Information). STEM analysis showed that the ruthenium particles in the KCC‐1‐NH_2_‐Ru samples were very well dispersed, with an average particle size of 1.2 ± 0.4 nm, with some particles as small as 0.4 nm, nearing atomic dispersion. For the Ru supported on silica spheres, SiO_2_–NH_2_–Ru, the dispersion was similar, with the mean particle size matching that of the KCC‐1‐NH_2_‐Ru samples (Figure S6, Supporting Information). Both samples therefore exhibit similar dispersion and loading of Ru, allowing for a direct comparison.

Previous reports indicate that alkali promotion is crucial to enhance the catalytic activity in NH_3_ decomposition. For this reason, prior to all the catalytic tests, samples were promoted with a 10 wt% K, unless otherwise indicated (see Supporting Information for experimental details).

The catalysts were tested in ammonia decomposition. As can be seen in Figure S7b, Supporting Information, KCC‐1‐NH_2_‐Ru‐K sample exhibited a local temperature close to 220 °C, while SiO_2_‐NH_2_‐Ru‐K reached 208 °C under the same reaction conditions.

**Figure 1:**
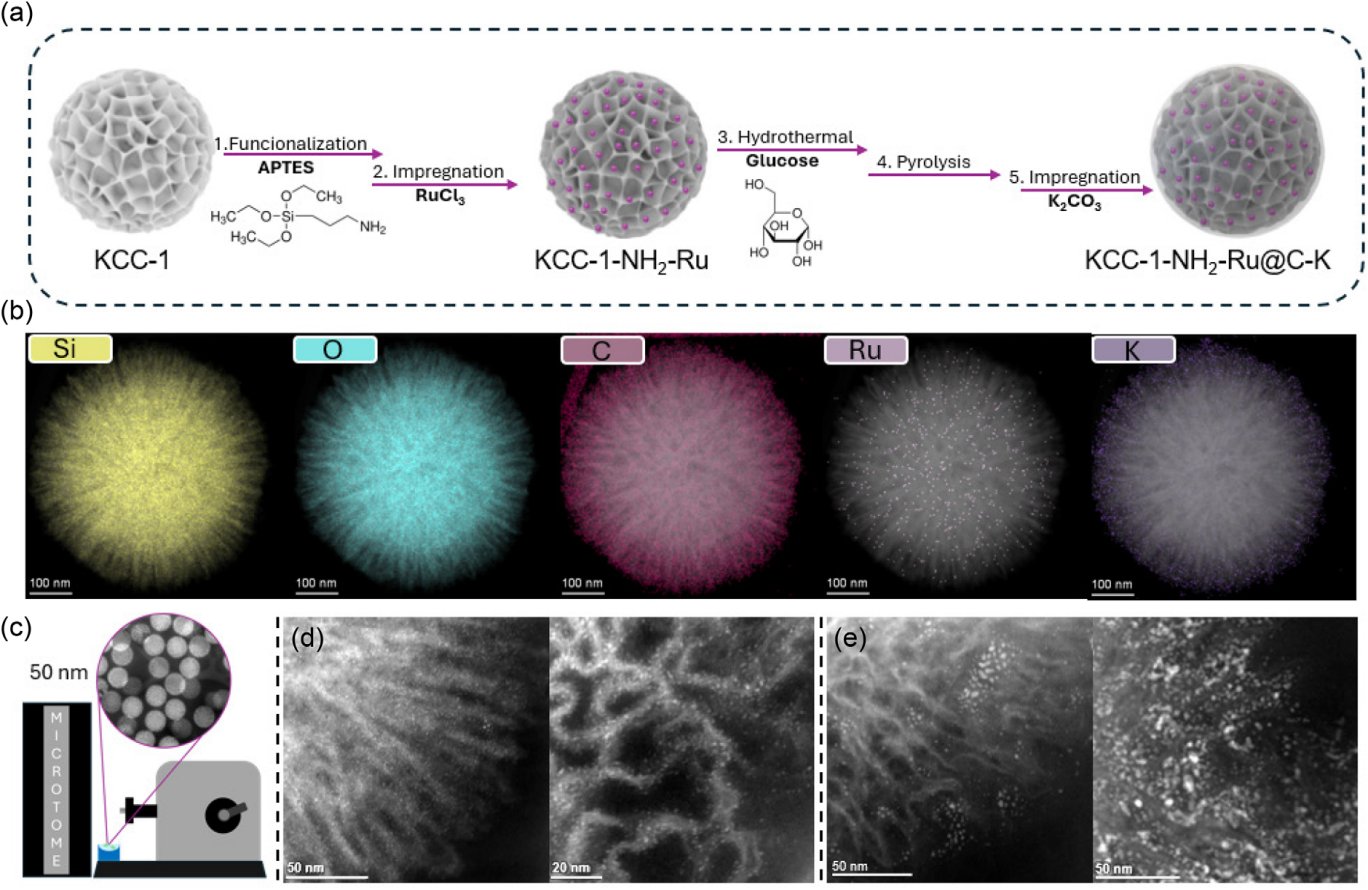
a) Scheme of synthesis of KCC‐1‐NH_2_‐Ru@C‐K. b) HAADF‐STEM images together with elemental mappings by EDX showing the presence of silicon (yellow), oxygen (blue), carbon (magenta), ruthenium (pink), and potassium (purple) in the sample KCC‐1‐NH_2_@C‐Ru‐K. c) Scheme of microtome cross‐section of 50 nm, and STEM images of ultramicrotome cross‐section of d) KCC‐1‐NH_2_‐Ru@C‐K and e) KCC‐1‐NH_2_@C‐Ru‐K.

To enhance the catalytic activity, we focused our efforts on optimizing the KCC‐1‐NH_2_‐Ru sample. Our approach involved coating this sample with a thin carbon layer to both increase light absorption and reduce infrared emission. This was achieved by suspending the KCC‐1‐NH_2_‐Ru in an aqueous glucose solution, followed by a hydrothermal treatment (sample denoted as KCC‐1‐NH_2_‐Ru@C‐K) (**Figure** **1**a). Detailed composition and structure information of both samples were further examined by scanning transmission electron microscopy (STEM) and elemental mapping analysis. The STEM–EDX mappings reveal the SiO_2_ core‐carbon shell structure and homogeneous distribution of potassium promoter (Figure 1b, S8, and S9b, Supporting Information). To verify the influence of the Ru encapsulation, we prepared an additional sample in which KCC‐1‐NH_2_ was first coated with carbon and then impregnated with Ru (sample denoted as KCC‐1‐NH_2_@C‐Ru‐K) (Figure S9a, Supporting Information). The nominal loading of Ru in both KCC‐1‐NH_2_‐Ru@C‐K and KCC‐1‐NH_2_@C‐Ru‐K was determined by TGA and ICP analysis, achieving a 1.65 wt% Ru (Table S1 and S2, Supporting Information). To determine the precise location of the Ru particles in both samples, we employed microtomy to section the catalyst particles into ultrathin slices of 50 nm (Figure 1c). Subsequently, we examined the samples using STEM, allowing us to capture cross‐sectional images of the catalyst particles. As depicted in Figure 1d, the Ru particles in the KCC‐1‐NH_2_‐Ru@C‐K sample were evenly distributed on the surface of the KCC‐1, displaying an average particle size of 1.0 ± 0.3 nm (Figure S10a, Supporting Information). Conversely, in the KCC‐1‐NH_2_@C‐Ru‐K sample, the Ru particles were positioned on the surface of the carbon, showing a certain aggregation that led to an average size of 2.6 ± 0.8 nm. (Figure 1e and S10b, Supporting Information).

It is important to highlight that the functionalization of KCC‐1 with amine groups was essential. Without this functionalization, the carbon would not adhere to the KCC‐1 particles, resulting in the formation of separate phases of KCC‐1 and carbon spheres (Figure S11, Supporting Information). This process also ensured a good dispersion of Ru particles on the KCC‐1 surface, which is crucial for their further encapsulation by the carbon. In fact, a blank sample without functionalization (KCC‐1‐Ru‐K) displayed very low catalytic activity and stability, probably due to the particle aggregation (Figure S7, Supporting Information). The light absorption properties of all samples were analyzed by UV‐vis‐NIR diffuse‐reflectance spectroscopy (Figure S12, Supporting Information). Not surprisingly, both KCC‐1 and KCC‐1‐Ru‐K displayed negligible light absorption in the visible and NIR regions owing to the insulating properties of the silica and the low Ru loading. Conversely, the samples containing the carbon coating exhibited an enhanced absorption of light from 300 up to 1500 nm, thus demonstrating the crucial role of the carbon shell as broad light absorber.

**Figure 2:**
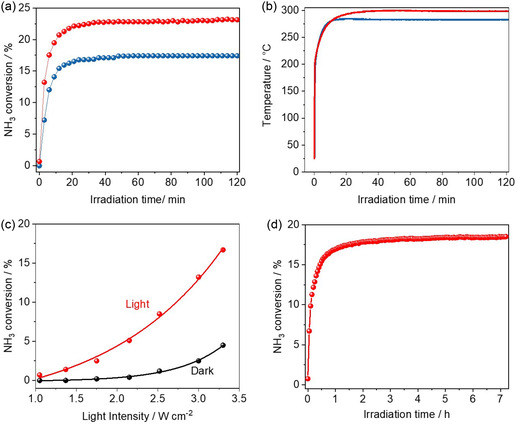
a) NH_3_ conversion as a function of the irradiation time for KCC‐1‐NH_2_@C‐Ru‐K (blue) and KCC‐1‐NH_2_‐Ru@C‐K (red). Reaction conditions: 3.5 W cm^−2^ b) Temperature profile for KCC‐1‐NH_2_@C‐Ru‐K (blue) and KCC‐1‐NH_2_‐Ru@C‐K (red) upon irradiation. c) Influence of the light intensity on the NH_3_ conversion of KCC‐1‐NH_2_‐Ru@C‐K under light (direct illumination, red line) and dark (indirect illumination, black line) conditions. d) Long‐term stability test of KCC‐1‐NH_2_‐Ru@C‐K. Reaction conditions: 3.3 W cm^−2^.

Samples were then tested in the photothermal ammonia decomposition, as illustrated in **Figure** **2**. Both catalysts demonstrated higher activity and displayed remarkably higher temperatures than those without carbon coating, highlighting the vital role of carbon in absorbing light and converting it into heat. Notably, the KCC‐1‐NH_2_@C‐Ru–K and KCC‐1‐NH_2_‐Ru@C‐K samples exhibited temperatures under irradiation as high as 280 and 300 °C, respectively. These elevated temperatures translated into remarkable H_2_ production rates in the order of 13,050 and 17,300 mmol gRu^−1^ h^−1^ for KCC‐1‐NH_2_@C‐Ru‐K and KCC‐1‐NH_2_‐Ru@C‐K, respectively. To the best of our knowledge, these values for catalytic NH_3_ decomposition outperform by far those from conventional Ru‐based thermal catalysts under low temperature (≤300 °C) and high space velocity (20,000 mL g^−1^ h^−1^) reaction conditions (see Table S5, Supporting Information). This catalytic activity is maintained for more than 7 h with non‐observable agglomeration of Ru or deactivation (Figure S13, Supporting Information).

We prepared a control sample, SiO_2_‐NH_2_‐Ru@C‐K, and observed that while it performed better than SiO_2_‐NH_2_‐Ru‐K (without the carbon coating), it still showed lower catalytic activity compared to KCC‐1‐NH_2_‐Ru@C‐K. This comparison further emphasizes the importance of using KCC‐1 as the support material, which, in combination with the carbon coating, leads to enhanced performance in ammonia decomposition (Figure S14, Supporting Information).

As we mentioned before, photo‐thermal catalysis arises from the synergistic combination of thermal and non‐thermal effects of light. A straightforward method to isolate both contributions is using an indirect illumination method, as described by Everitt and collaborators.^[^ ^38^ ^]^ In these experiments, a thin layer of Ti_2_O_3_ (a material with excellent properties to transform light into heat but inactive for the NH_3_ decomposition reaction) is placed on top of the catalyst bed to guarantee that the catalyst is not directly irradiated but displays the same temperature profile as in the photothermal reaction with direct illumination. Eventually, it is possible to examine the differences between the direct and indirect illumination experiments and obtain the relative contribution of pure photochemical effects to the overall catalytic activity. Figure 2c shows the influence of light intensity on the catalytic activity of both direct and indirect illumination experiments. As can be seen, the nonthermal contribution shows a positive relationship with the light intensity, particularly at high irradiances, thus indicating that the photochemical contribution is predominant. These observations are the typical signature of photothermal systems mainly dominated by nonthermal effects acting in combination with minor thermal effects. Nevertheless, it is worth reminding that the pure thermal contribution may be less significant but is essential to synergistically enhance the catalytic activity of the photochemical pathway. From these experiments it was also possible to calculate the apparent activation energy of the process under dark and light conditions (Figure S15, Supporting nformation), achieving values of 87.0 and 78.3 kJ mol^−1^, respectively, being in good agreement with the reported values in the literature for Ru‐based catalysts. This decrease in the activation energy under illumination indicates that the photo effect becomes present and more pronounced compared to the thermal effect under light. If only thermal effects were present, the activation energy would remain unchanged during illumination.

To further investigate the role of light in our system, we performed NH_3_‐TPD measurements both under light and dark conditions while monitoring the H_2_ and N_2_ generation through mass spectrometry (Figure S16, Supporting Information). These experiments revealed that the desorption of H_2_ molecules began at 276 °C under light radiation, whereas under dark conditions, H_2_ was detected only at temperatures above 300 °C. Surprisingly, when it comes to N_2_ desorption, light promoted the desorption of N_2_ at temperatures as low as 154 °C, roughly 50 °C lower temperature compared to dark conditions. Considering that for Ru‐based catalysts the recombinative desorption of N_2_ is generally considered the rate‐limiting step in the NH_3_ decomposition reaction, the here‐presented NH_3_‐TPD measurements are in complete agreement with the decrease in the activation energy observed under light radiation (vide supra).^[^ ^39^, ^40^, ^41^ ^]^ Altogether, these results demonstrate the crucial role of light to accelerate the desorption of H_2_ and N_2_, thus boosting the catalytic rate compared to dark conditions.

In order to provide additional evidences of the existence of nonthermal effects, we measured the photocurrent of the KCC‐1‐NH_2_‐Ru@C‐K sample under varying light intensities. As illustrated in Figure S17, Supporting Information, results indicate the presence of charge separation that depends on the radiation intensity, thereby reaffirming the photochemical contribution. To better understand the reaction mechanism, we also examined the photocurrent derived from KCC‐1‐NH_2_@C‐K and compared it with KCC‐1‐NH_2_‐Ru@C‐K and KCC‐1‐NH_2_@C‐Ru‐K samples (Figure S18, Supporting Information). As can be seen, the transient photocurrent of the sample without Ru showed a built up of charge indicative of an accumulation of charges trapped at the surface of the electrode. When the light was switched off, the current decrease was not instantaneous, but gradual, indicating a progressive discharge. This phenomenon has been previously described in N‐doped carbons that exhibit semiconducting properties thanks to the heteroatom doping.^[^ ^42^ ^]^ In our case, the amine functionalization would be responsible of the N‐doping of the carbon layer, so we hypothesize that the photocurrent of KCC‐1‐NH_2_@C‐K derives from the photoexcitation of the carbon shell. We attempted to measure the photocurrent of pristine KCC‐1‐Ru to verify the photoactivity of carbon but, unfortunately, the sample decomposed during photoelectrochemical tests, highlighting the protective role of carbon against the degradation of KCC‐1.^[^ ^42^ ^]^ Interestingly, our findings indicate that the incorporation of ruthenium nanoparticles leads to a reduction in the photocurrent response. This suggests a remarkable phenomenon wherein photoinduced charges undergo transfer from the carbon to the ruthenium nanoparticles that, eventually, act as charge trapping centers.^[^ ^43^ ^]^ To confirm this hypothesis, steady‐state photoluminescence studies were carried out (Figure S19, Supporting Information). As can be observed, the photoluminescence intensity decreased in the presence of ruthenium nanoparticles, demonstrating the existence of charge transfer events between the carbon and the Ru nanoparticles. Additional time‐resolved photoluminescence (TRPL) measurements were performed to evidence the photoinduced charge injection from the carbon to the Ru sites (Figure S20 and Table S3, Supporting Information). The KCC‐1‐NH_2_@C‐K sample exhibited an average decay time of 4.77 ns, however, samples containing Ru displayed an extended decay time in the order of 8.31 and 7.20 ns for the encapsulated and supported samples, respectively. These observations indicate that the photogenerated carriers can be trapped by Ru sites, leading to longer decay times. Overall, mechanistic experiments suggest that Ru active sites act as carrier traps to promote the NH_3_ decomposition reaction through the synergistic combination of nonthermal and thermal effects.

To evaluate the impact of carbon, we quantified the carbon content and analyzed its structural characteristics through thermogravimetric analysis and Raman spectroscopy (Figure S4 and S21, Supporting Information).^[^ ^44^ ^]^ The thermogravimetric analysis revealed that both samples, KCC‐1‐NH_2_‐Ru@C and KCC‐1‐NH_2_‐@C‐Ru, contained ≈30 wt% carbon. Raman spectroscopy confirmed that the structural arrangement of carbon was similar in both samples, as evidenced by identical D/G band ratios. These findings suggest that the carbon content and structure are consistent across samples, which is crucial since the microstructure of carbon influences its ability to convert light into heat. In view of this, the primary factor influencing their effectiveness as photothermal catalysts seems to be the position of the Ru particles, that is, embedded within the carbon matrix or situated on the surface.

To gain a better understanding of the system, we conducted a comprehensive characterization. The initial measurement involved X‐ray photoelectron spectroscopy. Figure S18, Supporting Information, displays the Ru 3*p* spectra for the various samples. Each sample exhibited a 3*p*_3/2_ doublet centered at 461.7 eV and a 3*p*_1/2_ at 484.06 eV, as well as another doublet at 3*p*_3/2_ (465.68 eV) and 3*p*_1/2_ (487.5 eV). The doublet at lower binding energies indicates reduced Ru, while the one at higher binding energies suggests oxidized Ru species. The proportion of both oxidized and reduced states was consistent in all samples, indicating that ruthenium is predominantly in the reduced state with a minor fraction in the oxidized form. This is due to the fact that during the synthesis of the material the Ru is partially oxidized. We also performed the quantification of the different components of the catalyst and found that in both samples KCC‐1‐NH_2_‐Ru@C‐K and KCC‐1‐NH_2_@C‐Ru‐K the surface composition is very similar and in line with TGA results (Figure S23 and Table S4, Supporting Information). Furthermore, temperature‐programmed reduction and N_2_ adsorption measurements indicated that both KCC‐1‐NH_2_‐Ru@C‐K and KCC‐1‐NH_2_@C‐Ru‐K samples show comparable redox properties and N_2_ adsorption capacities (Figure S5 and S24, Supporting Information).

**Figure 3:**
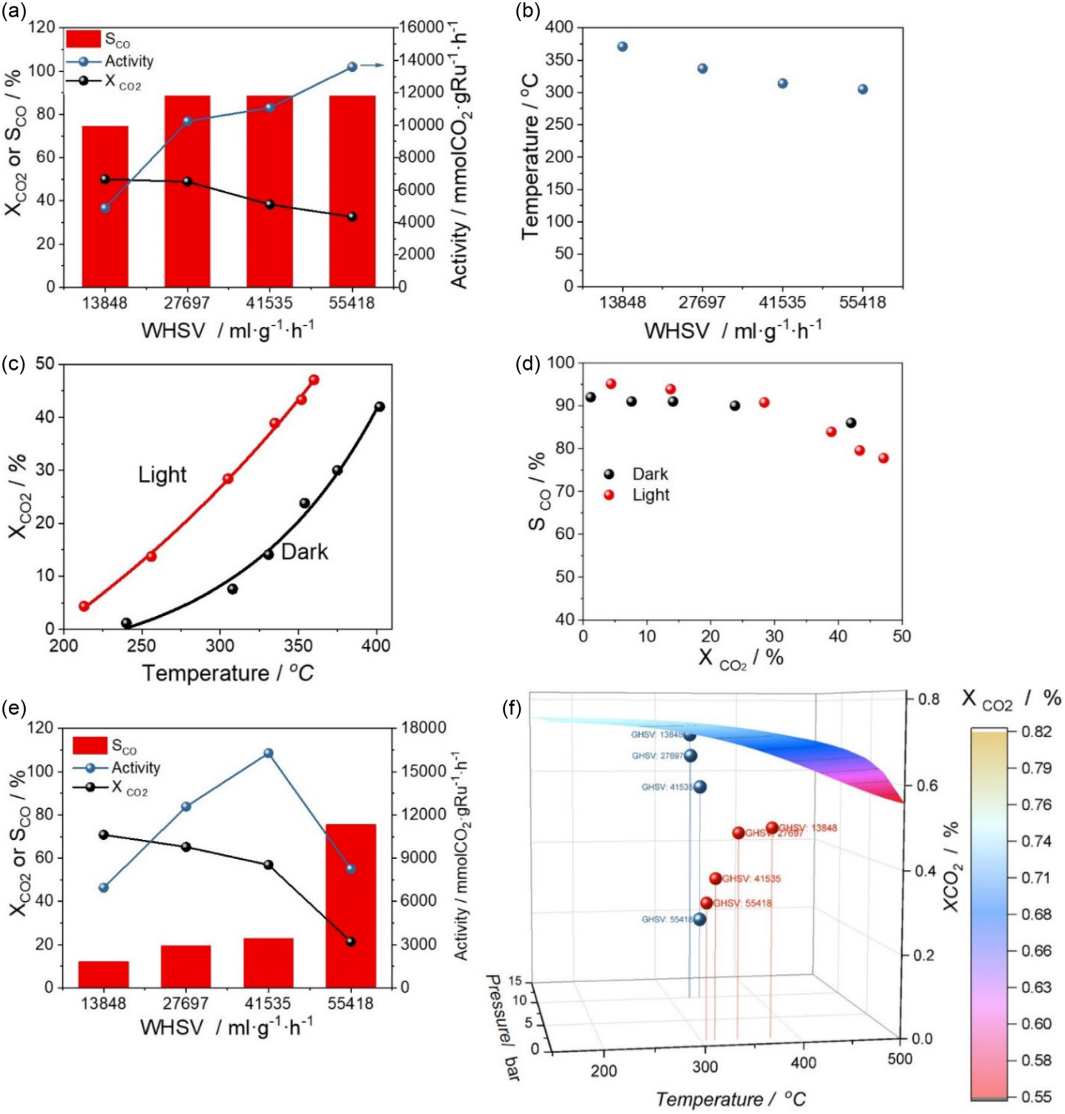
a) Catalytic performance of the KCC‐1‐NH_2_‐Ru@C sample in CO_2_ hydrogenation with different space velocities (WHSV). Reaction conditions: 1 bar b) Temperature reached by the sample under illumination at different WHSV. c) CO_2_ conversion at different temperatures under light (red) and dark (black) conditions. d) shows the CO selectivity versus CO_2_ conversion. Reaction conditions: 1 bar. e) Catalytic performance of the KCC‐1‐NH_2_‐Ru@C sample in CO_2_ hydrogenation at 10 bar with different space velocities (WHSV). f) Equilibrium conversion of CO_2_ at different pressures and temperatures, alongside the conversion achieved in the previous experiments.

Given the exceptional catalytic properties of the KCC‐1‐NH_2_‐Ru@C‐K sample in ammonia decomposition, we decided to use this catalyst in another reaction of interest, specifically CO_2_ hydrogenation. The catalyst was tested in CO_2_ hydrogenation using a CO_2_:H_2_ ratio of 1:3 at atmospheric pressure. Different space velocities were tested, and the results are shown in **Figure** **3**a. As expected, the conversion decreases with increasing space velocity. This reaction is equilibrium‐limited, so we calculated the thermodynamic equilibrium using Aspen software (Figure 3f). As it can be observed, the conversion values are below equilibrium, indicating that the catalysts are working in the kinetic regime. The catalytic activity, defined as the number of moles of CO_2_ converted per mass of active phase and time, increases with space velocity, thus revealing that the reaction is of positive order.

Ruthenium is renowned for its effectiveness as methanation catalyst. Nevertheless, our experiments showed an unexpected 90% selectivity toward CO at high space velocities (Figure 3a). We hypothesize that this outcome is due to the very small Ru particle size observed in our catalyst. In fact, various studies have demonstrated that Ru can function as a catalyst for the reverse water–gas shift (RWGS) reaction rather than methanation when the particles are very small or atomically dispersed. Kwak et al.^[^ ^12^, ^13^, ^45^ ^]^ found high CO selectivity in a fresh Ru/Al_2_O_3_ catalyst with isolated Ru atoms. During the CO_2_ hydrogenation process, methane became the main product, a result of the clustering of isolated Ru atoms into nanoparticles. Additionally, Aitbekova et al.^[^ ^12^, ^13^, ^46^ ^]^ discovered that oxidative pre‐treatment caused the redispersion of Ru nanoparticles on CeO_2_, converting a methanation catalyst into an RWGS catalyst.^[^ ^47^ ^]^ In our system, STEM images revealed that the Ru particles are extremely well‐dispersed and have a very small size, a fact that eventually would favor the RWGS reaction (Figure 2). When examining the temperature at which the catalyst heats up at each space velocity, we observe that this temperature decreases as space velocity increases (Figure 3b). This occurs because the gases enter the reactor cold, and as the flow rate increases, the cooling effect of the reactant flow becomes more pronounced at higher space velocities.

Similar to our study on ammonia decomposition, we investigated the effect of light on the catalytic activity for the CO_2_ hydrogenation reaction. To this end, we compared the CO_2_ conversion relative to the temperature achieved under different light intensities (Figure 3c). The results show that when heating by indirect illumination, higher temperatures are required to reach the same conversion level compared to direct illumination, with a temperature difference of 60–85 °C, depending on the conversion level. These observations correlate well with our previous results for NH_3_ decomposition and indicate, again, the presence of nonthermal effects in our system. Examining the selectivity and comparing it against conversion, we found that illumination does not affect the product generated. This suggests that the selectivity is primarily due to the small particle size.

Additionally, when we observe the shape of the curve, we notice that the conversion versus temperature curve becomes more linear under illumination. This implies that the photoinduced effects are starting to become evident. This trend in the curves is yet another indication of these photo effects, which were also indirectly analyzed using photocurrent and TRPL measurements.

We also examined the effect of pressure on this reaction by varying the space velocity and conducting experiments at 10 bar, as shown in Figure 3e. As expected, the conversion decreases with increasing space velocity. Notably, at the lowest space velocity, the conversion reaches a value very close to equilibrium (Figure 3f). This suggests that the catalyst is highly active at very low temperatures for this type of reaction. Table S6 and S7, Supporting Information, show the current state‐of‐the‐art of Ru‐based catalysts for both photothermal and thermal CO_2_ hydrogenation, demonstrating that our catalyst is one of the most actives known to date. Additionally, at 10 bar pressure, the selectivity shifts, with methane becoming the predominant product. This could be due to two factors: 1) at higher pressures, CO desorption from the surface is more difficult, leading to its hydrogenation to methane; and 2) we are operating close to the equilibrium conversion, where methane production is favored at these temperatures. Indeed, as we increase the space velocity and move away from equilibrium conversion, CO selectivity rises again, indicating that CO_2_ is initially hydrogenated via the RWGS reaction, and the generated CO is subsequently hydrogenated to methane.

It is important to note that all the experiments described in the CO_2_ hydrogenation study were conducted using the same sample and in the chronological order presented in the article. Each experiment lasted 5 h, and when considering all the experiments under different conditions and other optimization tests, the sample was tested for over 100 h without showing any sign of deactivation. In fact, to verify the sample's performance, we repeated the first experiment, testing the used sample at atmospheric pressure with a gas hourly space velocity of 13,848 mL g^−1^ h^−1^. Under these conditions, a CO_2_ conversion of 50.5% and a CO selectivity of 73% were obtained, being in good agreement with our initial results. This demonstrates, again, the excellent stability of this catalyst.

We tested the SiO_2_‐NH_2_‐Ru@C‐K in the CO_2_ hydrogenation reaction, achieving low CO_2_ conversion values of 11% compared to the 75% conversion obtained with the KCC‐1‐NH_2_‐Ru@C‐K sample. This result confirms, again, the positive effect of KCC‐1 in the catalytic activity of the composite catalyst.

## 3 Conclusion

The results of this research confirm that the KCC‐1‐NH_2_‐Ru@C composite catalyst is highly effective for photothermal catalysis, offering significant advantages in terms of activity and durability. The successful encapsulation of ruthenium nanoparticles within a carbon layer on a mesoporous silica support ensures optimal light absorption. Overall, mechanistic experiments suggest that Ru active sites act as carrier traps to promote chemical reactions through the synergistic combination of nonthermal and thermal effects. This study paves the way for the development of advanced photothermal catalysts with tailored optical and thermal properties for renewable energy processes, emphasizing the importance of material design in achieving superior catalytic performance.

## Conflict of Interest

The authors declare no conflict of interest.

## Supporting information

Supplementary Material
