# Supplementary material for: Carbon‐Coated Urchin‐Like Silica Nanospheres for Enhanced Photothermal Catalysis
Source: ChemSusChem. 2025 Apr 8;18(12):e202500068. doi: 10.1002/cssc.202500068 (PMC12175052; doi:10.1002/cssc.202500068)
Supplement: Supplementary file 1 — Supplementary Material [file CSSC-18-e202500068-s001.pdf]

Supporting Information

Carbon-coated Urchin-like Silica Nanospheres for Enhanced Photothermal Catalysis

Alejandra Rendon-Patino[a], Xinhuilan Wang[a], S. Duran-Uribe[b], A. Sepulveda-Escribano[b], Diego Mateo[a,], Enrique V. Ramos- Fernandez[a,b,*] and J. Gascon[a,*].*

[a] KAUST Catalysis Center, King Abdullah University of Science and Technology, Thuwal, 23955-6900, Saudi Arabia

[b] Laboratory of Advanced Materials, Inorganic Chemistry Department, University Materials Institute of Alicante, University of Alicante, Apartado 99, Alicante, E-03080, Spain

* Enrique V. Ramos Fernández, email: enrique.ramos@kaust.edu.sa

Experimental Procedures

Materials and reagents

D-(+)-Glucose (C6H12O6, CAS 50-99-7), Potassium carbonate (K_2CO_3, CAS 584-08-7) , Ruthenium(III) chloride hydrate ($RuCl_3 \cdot xH_2O$, CAS 14898-67-0), urea (CH_4N_2O, CAS 57-13-6), hexadecyltrimethylammonium bromide (CTAB, $[(C_{16}H_{33})N(CH_3)_3]Br$, CAS 57-09-0), tetraethyl orthosilicate (TEOS, $Si(OC_2H_5)_4$, CAS 111-72-31-2), 1-butanol ($C_4H_{10}O$, CAS 71-36-3), toluene ($C_6H_5CH_3$, CAS 108-88-3) and 3-aminopropyltriethoxysilane(APTES, $H_2C{=}C(CH_3)CO_2(CH_2)_3$ $Si(OCH_3)_3$, CAS 2530-85-0) were purchased from Sigma-Aldrich. Ethanol (C_2H_5OH, CAS 64-17-5) was purchased from Fisher Scientific and deionized water was obtained with a Milli-Q® system (18.2 MΩ·cm).

Synthesis

Synthesis of SiO_2 spheres

Silicon oxide spheres were prepared using the Stöber hydrolysis procedure and used as templates. In a 500 mL flask, 17 mL of Milli-Q water, 4 mL of NH_4OH, and 153 mL of ethanol were added. This mixture was stirred for 30 min at 500 rpm and 50 °C. Subsequently, 22 mL of TEOS was added, and the mixture was maintained under the same conditions for 24 h. After this process, the solution was centrifuged for 30 min at 14,000 rpm. Finally, the sample was dried in an oven at 100°C and then calcined, with the temperature increased at a rate of 10°C·min up to 500°C, and held at this temperature for 30 min.

Synthesis of KCC-1

Dendritic fibrous KCC-1 was synthesized using a Professor Basset's method. Initially, 14.9 g of urea and 23.8 g of CTAB were mixed in 737.7 mL of distilled water, with stirring for 30 min at ambient temperature. Simultaneously, a solution containing 63.4 g of tetraethyl orthosilicate, 31.2 mL of 1-butanol, and 731.8 mL of toluene was prepared by mixing for 20 min at room temperature. The two solutions were then combined and stirred together for another 30 min at room temperature. The resulting mixture was transferred into a Teflon vessel and exposed to 400 W of intermittent microwave radiation for 4 h at 120°C.

After cooling to room temperature, the mixture was air-dried at 110°C. Finally, the white precipitate obtained was calcined at 800°C for 6 h to produce the dendritic fibrous KCC-1 catalyst.

Synthesis KCC-1-NH_2 and SiO_2-NH_2

0.02 g of KCC-1 or SiO_2 was dispersed in 1.2 mL of dried toluene and subjected to 30 min of sonication. Subsequently, 50 µL of APTES was added to the dispersion, and the mixture was refluxed for 20 h at 80°C. Following the reaction, the resulting mixture was washed multiple times with ethanol to remove any residual reactants. Finally, the functionalized material was dried at 80°C overnight.

Synthesis KCC-1-NH_2-Ru@C (encapsulated)

200 mg of KCC-1-NH_2 was impregnated with an aqueous solution of $RuCl_3$ (2 % wt.) using the pore volume method. Following impregnation, the sample was dried at 60°C for 30 min.

Subsequently, the dried sample was subjected to pyrolysis, during which it was gradually heated at a rate of 5 °C min^{-1} until reaching 350 °C, and holding it at this temperature for 2 h.

Following this, KCC-NH_2-Ru was mixed with 6 mL of an aqueous solution of glucose (1.25 g mL^{-1}). Then, it was introduced into a 10 mL autoclave for hydrothermal treatment (180 °C·min^{-1}, 3h). After the autoclave cooled to room temperature, the obtained solid was washed several times with a mixture of water and ethanol. After drying overnight, it was pyrolyzed in a nitrogen atmosphere with a ramp of 10 °C·min^{-1}, at 600 °C for 2 h.

Synthesis KCC-1-NH_2@C-Ru (supported)

200 mg of KCC-1-NH_2 was combined with 6 mL of an aqueous glucose solution (1.25 g mol^{-1}). The mixture was placed in a 10 mL autoclave and subjected to hydrothermal treatment at 180 °C for 3 h. After the autoclave was cooled to room temperature, the resulting solid was washed several times with a mixture of water and ethanol. The washed solid was dried overnight and then pyrolyzed

in a nitrogen atmosphere with a ramp rate of 10 °C·min^{-1}, heating to 600°C and holding this temperature for 2 h.

200 mg of KCC-1-NH_2@C was impregnated with an aqueous solution of $RuCl_3$ (1.65 % wt.) using the pore volume method. Following impregnation, the sample was dried at 60°C for 30 min. Subsequently, the dried sample was pyrolyzed, during which it was gradually heated at a rate of 5 °C min^{-1} until reaching 350 °C, and holding it at this temperature for 2 h.

Synthesis KCC-1-Ru

The KCC-1 support material was impregnated with a 1.65 % solution of Ruthenium precursor using a volumetric pore impregnation method. Subsequently, the impregnated material was reduced under a nitrogen atmosphere following a specific oven program: ramping the temperature at a rate of 5 °C ·min-^{1} up to 350 °C and holding it at this temperature for 2 h.

Potassium promotion Step

In preparation for the ammonia decomposition and RWGS tests, all samples were subjected to a standardized impregnation process with a 10 %wt. potassium precursor. Subsequently, they were treated at 350°C for 2 h with a ramp of 5 °C min^{-1} in a nitrogen atmosphere.

. Characterization

Scanning electron microscopy (SEM) images were obtained with Merlin (ZEISS, Germany) at a voltage of 5 kV. Transmission. For SEM analysis, the samples were fixed to carbon tape and coated with a 3 nm thick iridium layer supplied by Ted Pella. Infrared (IR) spectra were obtained utilizing an FT-IR Magna spectrometer 550 Nicolet, with analysis conducted on KBr plates.

Thermogravimetric (TG) data were collected in an air atmosphere using a Mettler-Toledo instrument (Mettler Toledo, USA) with a heating rate of 5 °C min^{-1}. The measurements were conducted in the temperature range of 25-800 °C with a gas flow of 25 mL min^{-1}.

N_2 adsorption-desorption measurements were performed at 77 K using a Micromeritics ASAP 2040 instrument (Micromeritics, USA). Before the

measurements, the samples were degassed at 120 °C for 12 h and at 180 °C for 16 h under vacuum.

X-ray photoelectron spectroscopy (XPS) measurements were performed on a K-Alpha spectrophotometer (Thermo-Scientific) with a high-resolution monochromator. All spectra were measured using monochromatized Mg-Kα radiation by a double crystal monochromator with the hemispherical analyser. The binding energies were calibrated using the 1s transition of carbon (C 1s, 284.60 eV). For compositional profile measurements, etching was performed using the high current mode with an Ion energy of 2000 eV for 60 seconds at each point.

H_2-TPR measurements were analyzed using a Micromeritics AutoChem 2920 unit. 70 mg catalyst were preheated with helium at 150°C for 30min, then cooled down to 50 °C. Subsequently, the catalyst was heated in a 50 $mL \cdot min^{-1}$ flow of 10 % H_2/Ar to 800 °C with a heating rate of 10 $°C \cdot min^{-1}$.

NH_3 temperature-programmed desorption (NH_3-TPD) analyses were performed using a Harrick Praying Mantis High-Temperature reaction chamber. 60 mg of the catalyst were initially preheated at 150 °C for 30 minutes under Ar flow. Subsequently, the sample was exposed to a flow of 50 vol% NH_3/Ar for 60 minutes and then flushed with argon for 30 minutes at 50 °C to remove the physical adsorbed NH_3. Then NH_3-TPD measurement was performed by recording the corresponding gas phase evolution via INFICON Transpector CPM100 mass spectrometry (MS) while heating the catalyst from 50 °C to 500 °C at a rate of 5 °C min^{-1} under an argon flow. To investigate the effect of illumination on NH_3 decomposition, light was introduced into the reaction chamber during the NH_3 desorption using a white LED source (UHP-T-WCS-DI, Prizmatrix)

Scanning electron microscopy (SEM) images were obtained with Merlin (ZEISS, Germany) at a voltage of 3 kV. Transmission. For SEM analysis, the samples were fixed to carbon tape and coated with a 3 nm thick platinum layer supplied by Ted Pella.

Transmission electron microscopy (TEM) images were obtained on a Titan ST (FEI Company, USA) at 300 keV. In particular, all STEM samples were prepared on dry copper grids with a Formvar film coated with a thin carbon layer (300 mesh).

Scanning transmission electron microscopy-high angle annular darkfield (STEM-HAADF) images were obtained on a Titan Cs-probe corrected Titan microscope (FEI Company, USA) at 300 kV. Samples for cross-section images were prepared by embedding the sample in Epofix Resin (Agar Scientific). After drying the resin at room temperature for 3 days, the pellet sample was cut into sections ranging from 70 to 90 nm using a Leica RM2255 ultramicrotome (Leica, Germany) and collected on 200-mesh copper grids.

Raman spectra were collected using a Witec Alpha 300 confocal Raman microscope system equipped with a RayShield coupler and a 532 nm laser as the excitation source. Spectra were acquired with a 50× objective working at a laser power of 2 mW, with an exposure time of 10 s and with 40 accumulations.

UV-vis-NIR optical diffuse reflectance spectra were collected using a Lambda 950 spectrophotometer with an integrating sphere in the acquisition range of 300 – 1500 nm at room temperature. $BaSO_4$ was used as reference.

Steady-state photoluminescence spectra were recorded in a Carry Eclipse Fluorescence Spectrometer using an excitation wavelength of 374 nm.

Time-resolved photoluminescence (TRPL) spectra were measured using a FluoroMax-4 spectrometer. The catalyst was dispersed in deionized water and sonicated to achieve a uniform dispersion (0.5 mg/mL). Then 2 mL of the dispersion was then transferred into a cuvette and degassed under N_2 for 30 minutes. Decay transients were recorded with an excitation wavelength of 375 nm and an emission wavelength of 485 nm.

Photoelectrochemical measurements were conducted in a 3-electrode electrochemical cell. The reference electrode was Ag/AgCl, the counter electrode was platinum, and the working electrode was composed of KCC-NH_2@C, KCC-NH_2@C-Ru and KCC-NH_2-Ru@C deposited on fluorine-doped tin oxide (FTO). The measurements were carried out using a SP-150 potentiostat (Bio-Logic). A 0.1 M solution of KCl served as the electrolyte, and during the measurements, N_2 was bubbled through the solution to ensure complete saturation. All the working electrodes were prepared by applying a 1% Paraloid solution in acetone as the binder, followed by drying at 50 °C.

The NH3 temperature-programmed desorption (NH3-TPD) analyses were performed using the Kubelka-Munk mode on a Thermo Scientific Nicolet 6700 series Fourier transform infrared (FT-IR) spectrometer equipped with a Harrick

Praying Mantis High-Temperature reaction chamber. Before collecting the background, the sample (KCC-1/KBr = 1:40) was preheated with a flow of N2 at 150 °C for 30 minutes. The background spectra were collected with 248 scans at 4 cm^{-1} resolution when sample cooled down to 50 °C. After that, the sample was exposed to a flow of 5%NH3/N2 for 60 minutes and then flushed with N2 for 30 minutes at 50 °C. Subsequently, the temperature was increased from 50 to 600 °C at a ramping rate of 5 $°C·min^{-1}$ under a flow of N2. During this stage, IR spectra were recorded with 64 scans at 4 cm^{-1} resolution every 2 min.

Photo-thermal experiments

Photo-thermal experiments were conducted using a continuous-flow setup equipped with a commercial Harrick® cell, modified with a custom-made quartz window to accommodate the specific experimental requirements.

In the case of NH_3 decomposition experiments, 30 mg of catalyst was carefully placed into the reactor cup. Prior to the commencement of irradiation, the reaction chamber was purged with a flow of NH_3 at a rate of 10 mL · min^{-1} for 15 min. Illumination was provided by a Xe lamp (Peccell Technologies) positioned a 4 cm above the reactor, ensuring uniform light distribution across the sample. The evolved gases from the reaction were continuously analyzed using a micro gas chromatograph (microGC, SRA Instruments). The microGC was directly connected to the reactor outlet, enabling real-time monitoring and analysis of the gas composition.

In the RWGS (Reverse Water-Gas Shift) experiments, 40 mg of the prepared catalyst was loaded into the reactor cup. Prior to irradiation, the reaction chamber was purged with the reaction mixture for 15 minutes, utilizing a flow of 10 $mL·min^{-1}$ of a CO_2 and H_2 mixture (with a CO_2 ratio of 1:3) and 1 $mL·min^{-1}$ of N_2. A Xe lamp (Perfect Light), positioned 5 cm above the reactor, served as the irradiation source. The evolved gases were analyzed by a gas compact chromatograph (CompactGC, Thermo Fisher) directly connected to the reactor outlet.

In both experimental setups, temperature monitoring during illumination was achieved using a thin thermocouple positioned just 1 mm below the catalyst

surface. This placement allowed for precise measurement of temperature changes directly influenced by the photo-thermal effect.

Supporting Figures

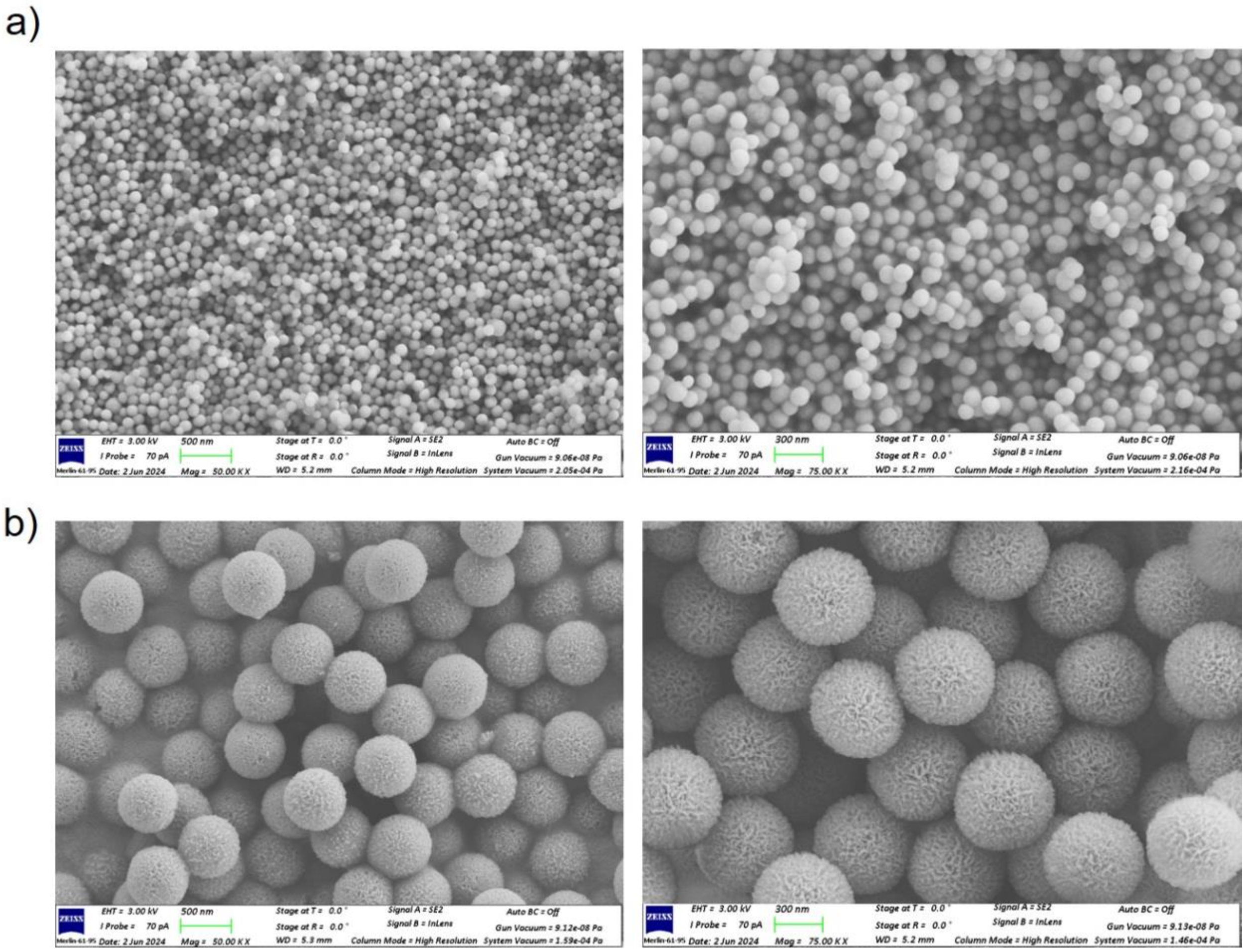

Figure S1. SEM images of a) KCC-1 and b) SiO_2 spheres.

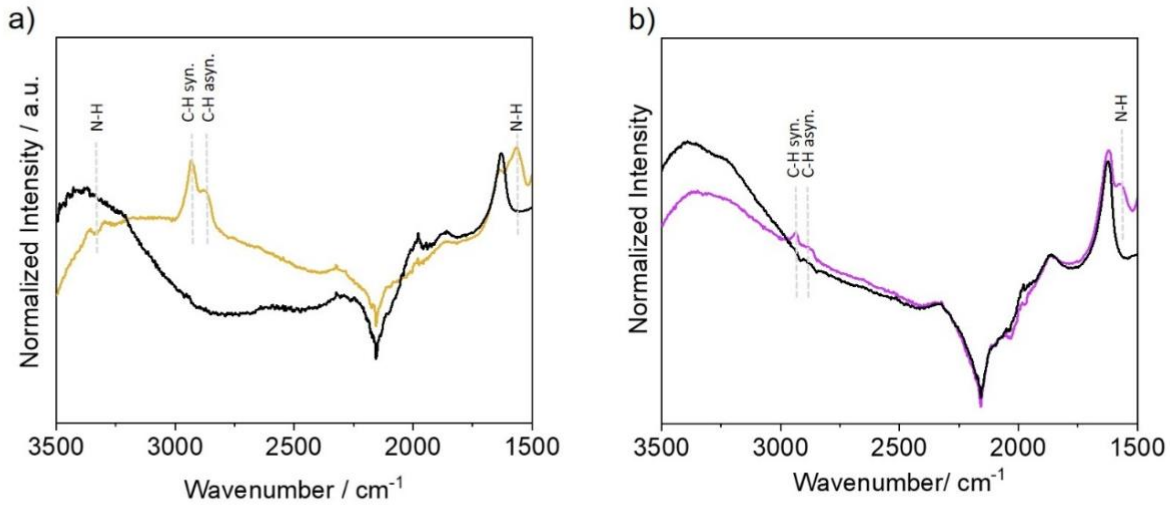

Figure S2. Infrared spectra of a) KCC-1 (black) vs. KCC-1-NH_2 (yellow), and b) SiO_2 (grey) vs. SiO_2-NH_2 (magenta).

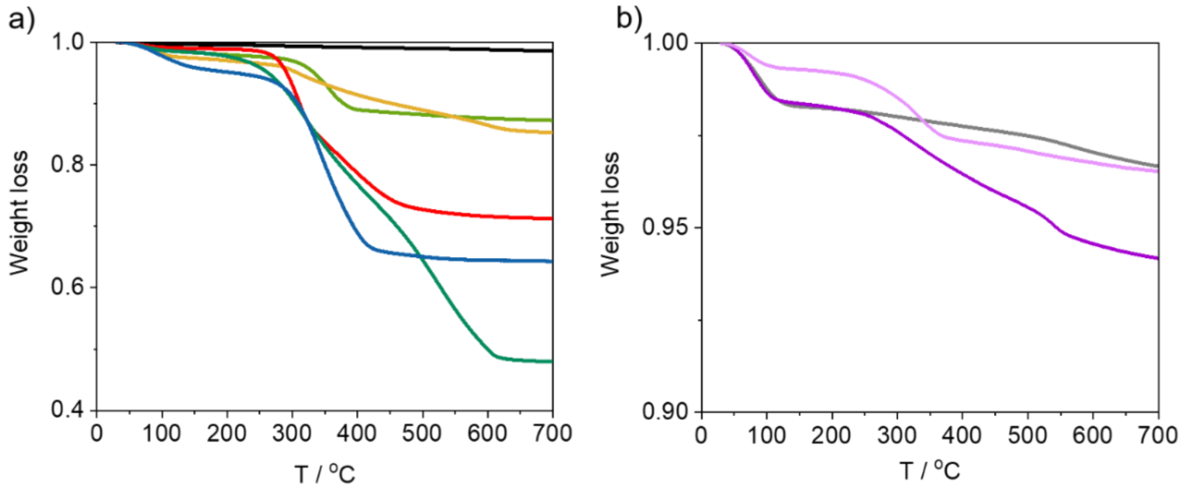

Figure S3. TGA profiles under air of various samples: a) KCC-1 (black), KCC-1-NH_2 (yellow), KCC-1-NH_2-Ru (light green), KCC-1-NH_2-Ru@C (red), KCC-1-NH_2@C (green), and KCC-1-NH_2@C-Ru (blue); b) SiO_2 (grey), SiO_2-NH_2 (magenta) and SiO_2-NH_2-Ru (pink).

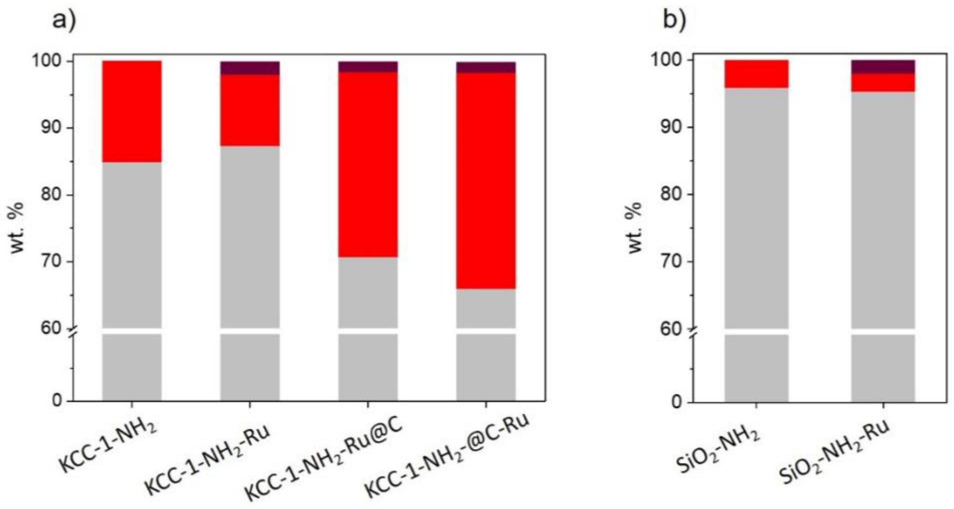

Figure S4. Calculated weight percentages of Ruthenium (maroon), Carbon (red), and Silicon oxide (grey) based on TGA measurements. For calculations it was considered that Ru remains as RuO_2.

Table S1. Ruthenium weight percentage from TGA analysis.

Sample	Ruthenium (%)
**SiO_2-NH_2-Ru**	1.68
**KCC-1-Ru**	1.55
**KCC-1-NH_2-Ru-K**	1.69
**KCC-1-NH_2@Ru-K**	1.59
**KCC-1-NH_2-Ru@C-K**	1.60

Table S2. Ruthenium weight percentage from ICP measurements.

Sample	Ruthenium (%)
**SiO_2-NH_2-Ru**	1.42
**KCC-1-Ru**	1.34
**KCC-1-NH_2-Ru-K**	1.47
**KCC-1-NH_2@Ru-K**	1.43
**KCC-1-NH_2-Ru@C-K**	1.65

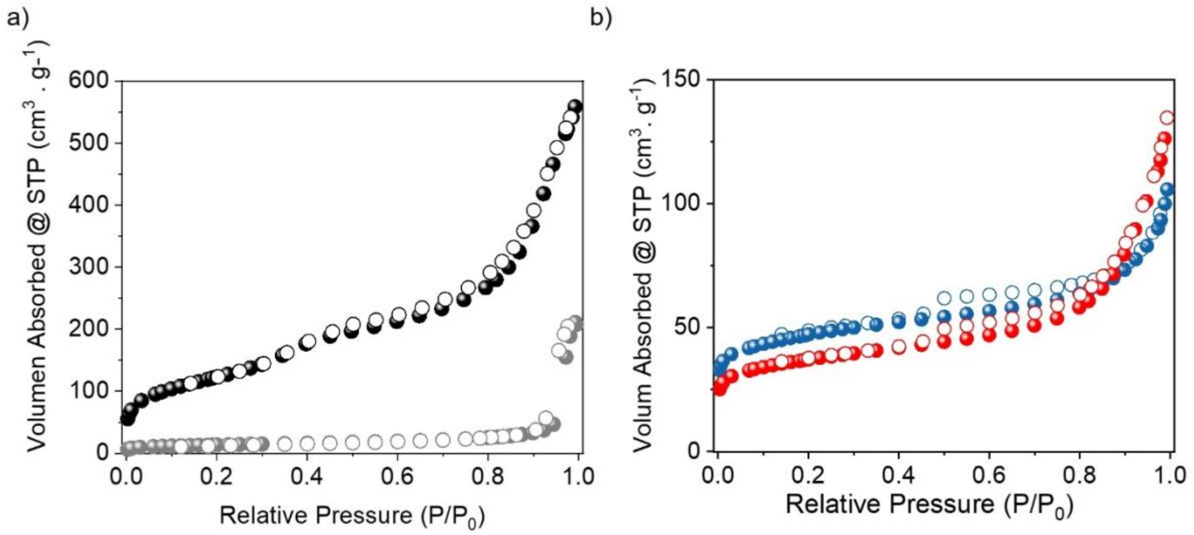

Figure S5. Nitrogen adsorption-desorption isotherms at 77 K for the samples: a) KCC-1 (black) and SiO_2 (gray); b) KCC-1-NH_2-Ru@C-K (red) and KCC-1-NH_2@C-Ru-K (blue). Filled symbols represent adsorption isotherms, and empty symbols represent desorption isotherms.

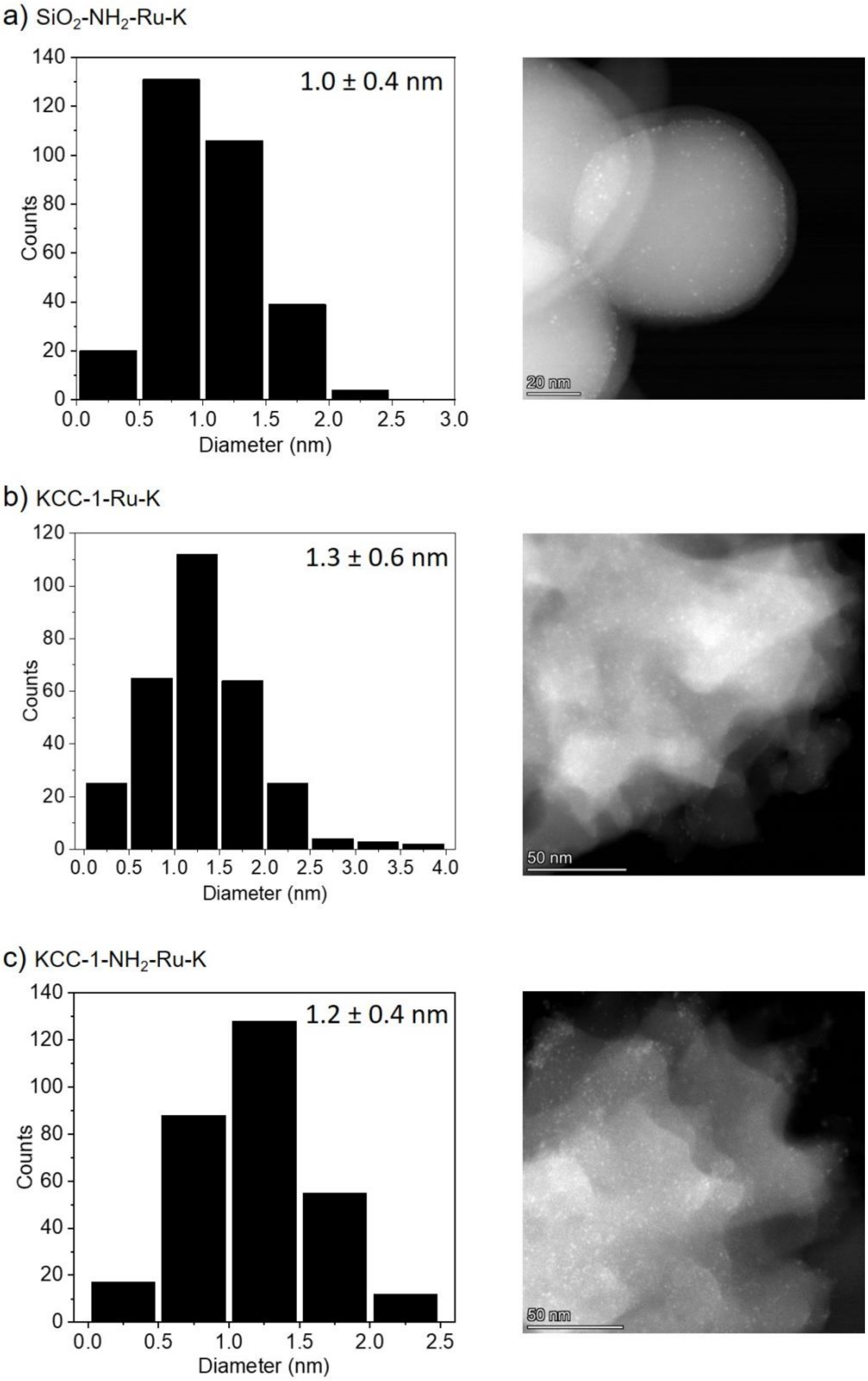

Figure S6. Particle size distribution and STEM images of a) SiO_2-NH_2-Ru-K, b) KCC-1-Ru and-K c) KCC-1-NH_2-Ru-K.

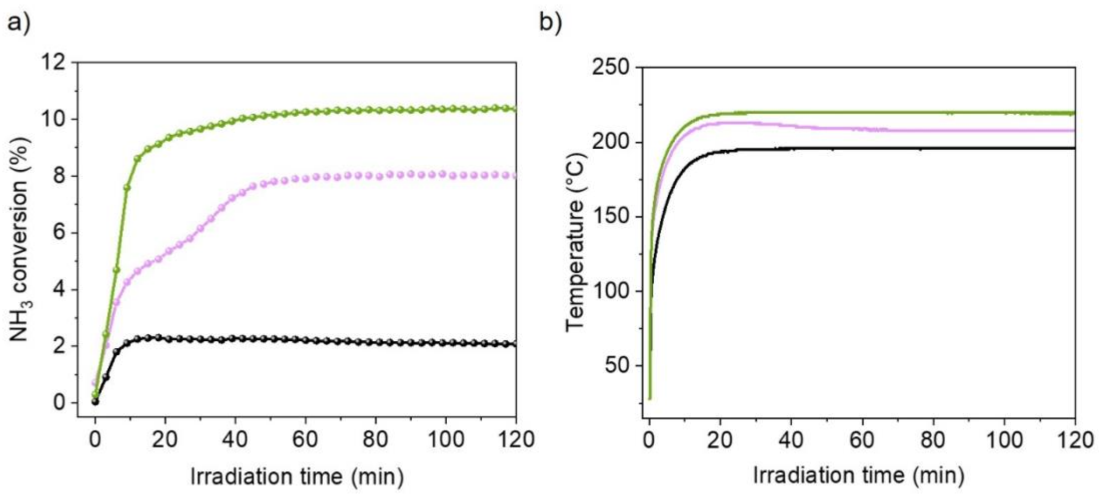

Figure S7. a) Conversion profiles and b) temperature profiles for the photo-thermal NH_3 decomposition reaction using KCC-NH_2-Ru-K (yellow), SiO_2-NH_2-Ru-K (magenta) and KCC-1-Ru-K (black).

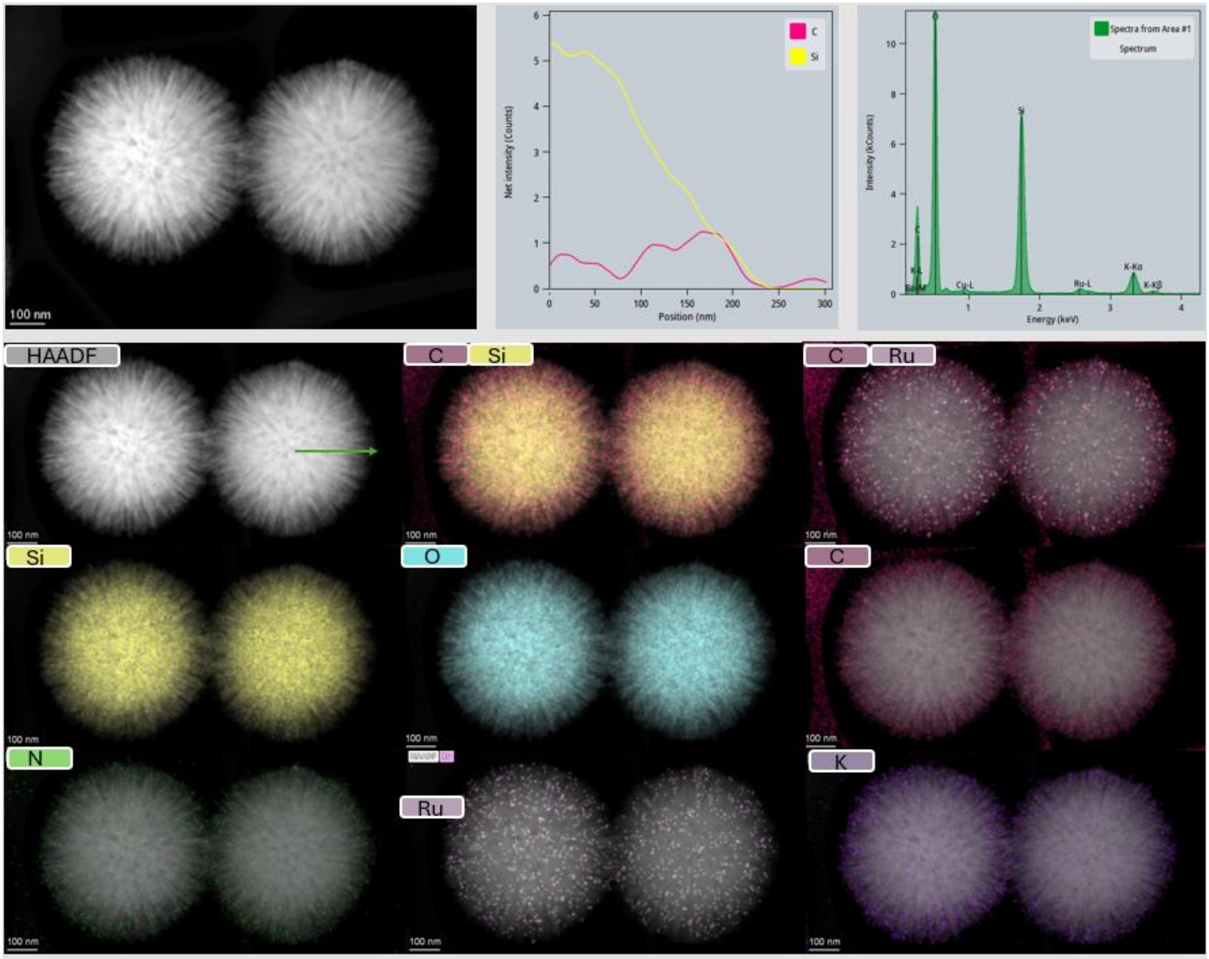

Figure S8. HAADF-STEM images together with elemental mappings and line analysis by EDX showing the presence of carbon (magenta), silicon (yellow), ruthenium (pink), nitrogen (green) oxygen (blue) and potassium (purple) in the sample KCC-1-NH_2-Ru@C-K.

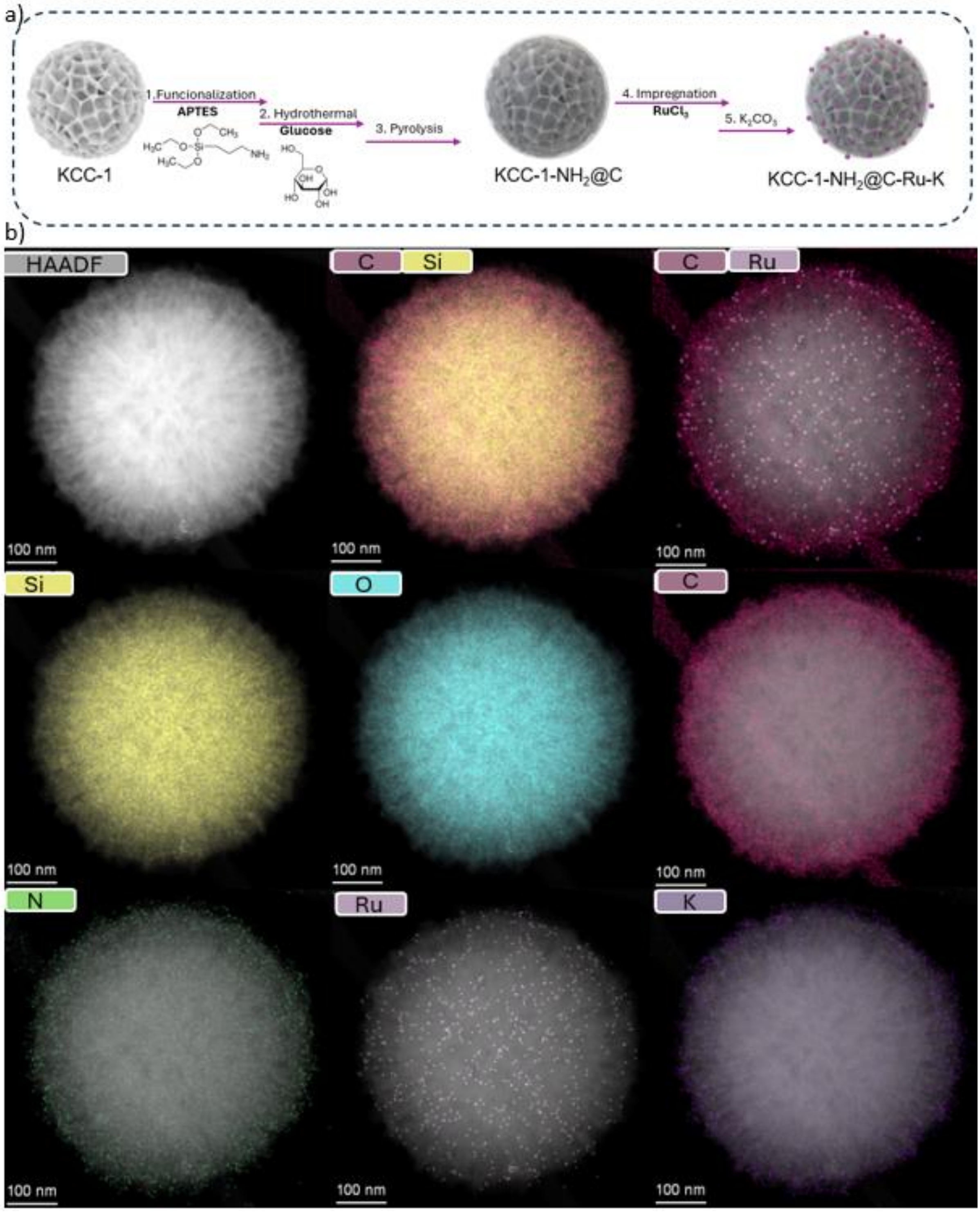

Figure S9. a) Scheme of synthesis of KCC-1-NH_2@C-Ru-K. b) HAADF-STEM images together with elemental mappings by EDX showing the presence of carbon (magenta), silicon (yellow), ruthenium (pink), nitrogen (green) oxygen (blue) and potassium (purple) in the sample KCC-1-NH_2@C-Ru-K.

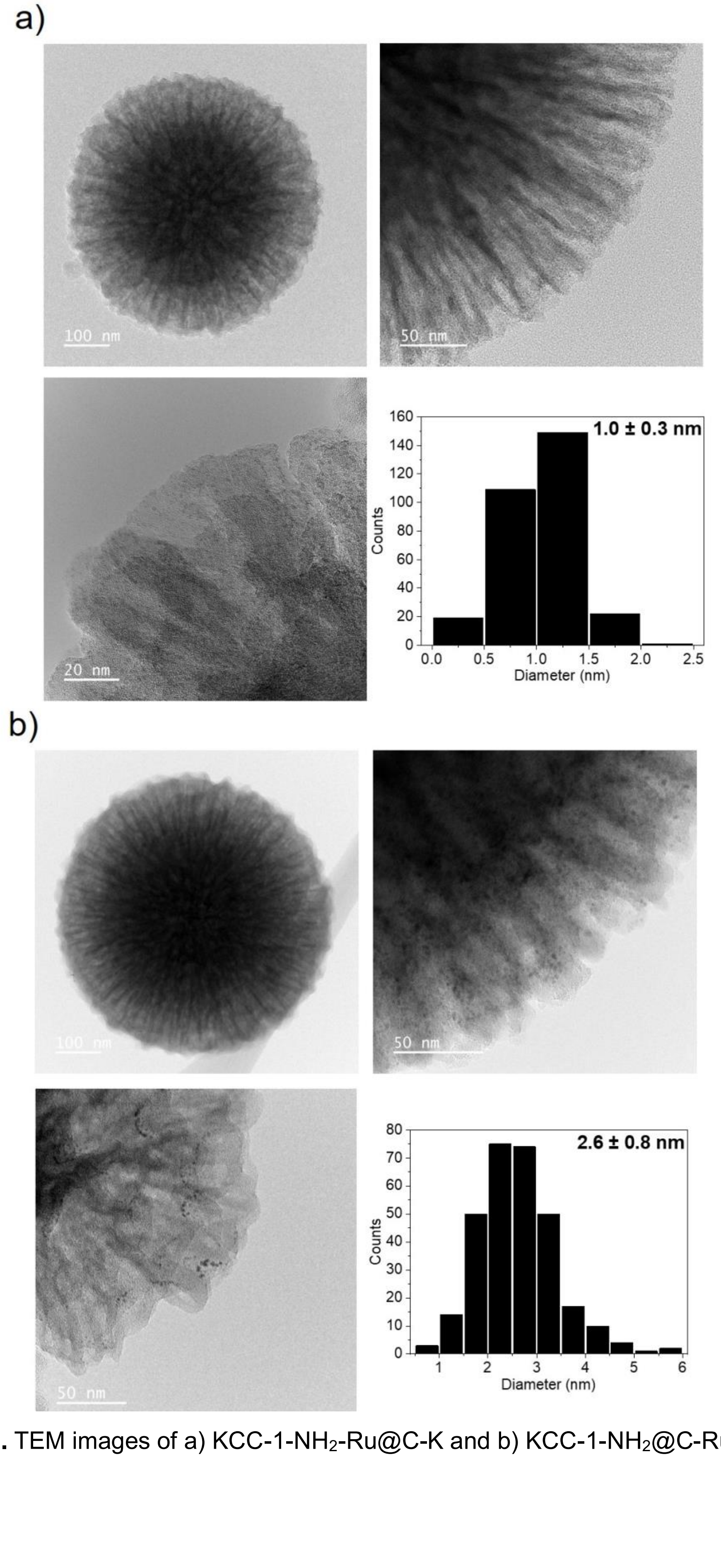

Figure S10. TEM images of a) KCC-1-NH_2-Ru@C-K and b) KCC-1-NH_2@C-Ru-K.

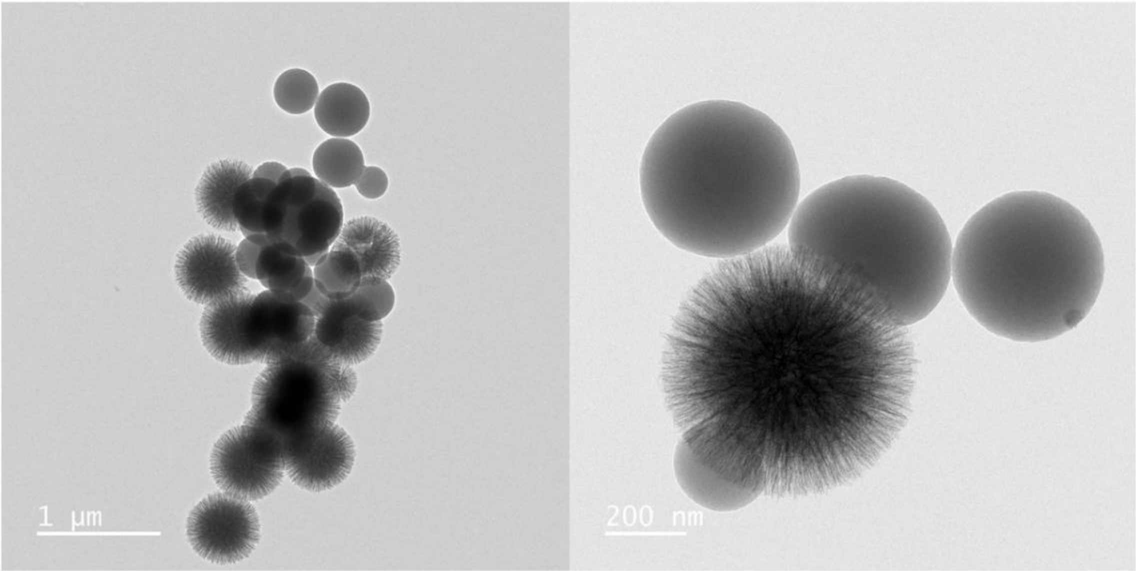

Figure S11. HRTEM images of KCC-1 and carbon spheres. In the absence of amino functionalization of KCC-1, carbon grows in a separate phase in the form of spheres.

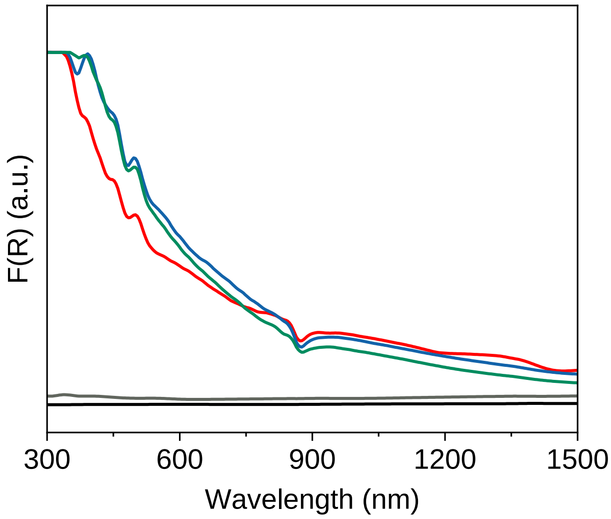

Figure S12. UV–visible-NIR diffuse reflectance spectra of KKC-1 (black), KCC-1-Ru-K (grey), KCC-1-NH_2-@C-K (green), KCC-1-NH_2@C-Ru-K (blue) and KCC-1-NH_2-Ru@C-K (red).

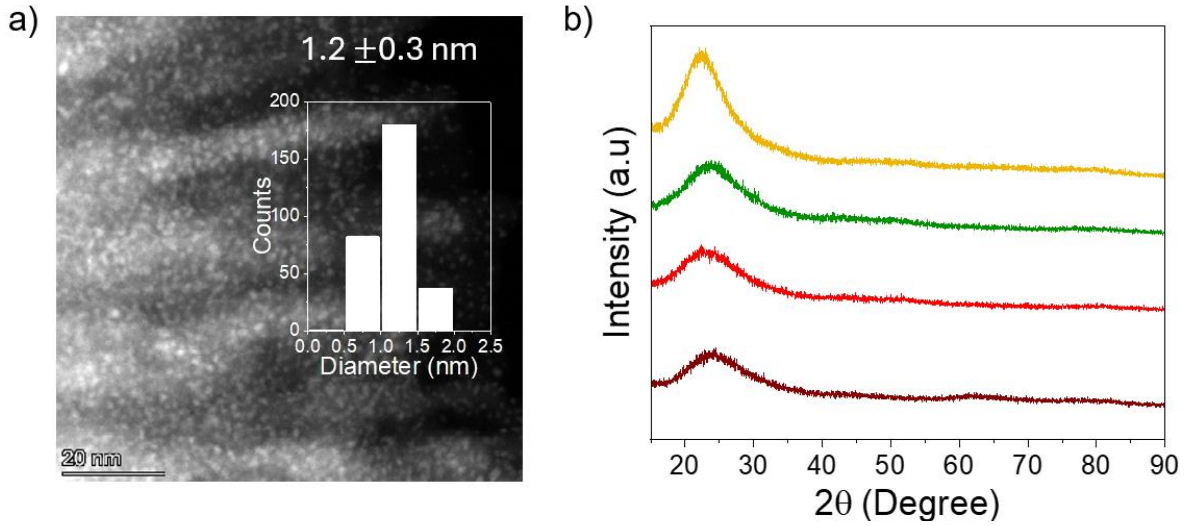

Figure S13. a) Particle size distribution and STEM image of KCC-1-NH_2-Ru@C-K after stability test. b) XRD pattern of : KCC-1-NH_2 (yellow), KCC-1-NH_2@C-K (green), KCC-1-NH_2-Ru@C-K (red) and KCC-1-NH_2-Ru@C-K after stability test. (maroon).

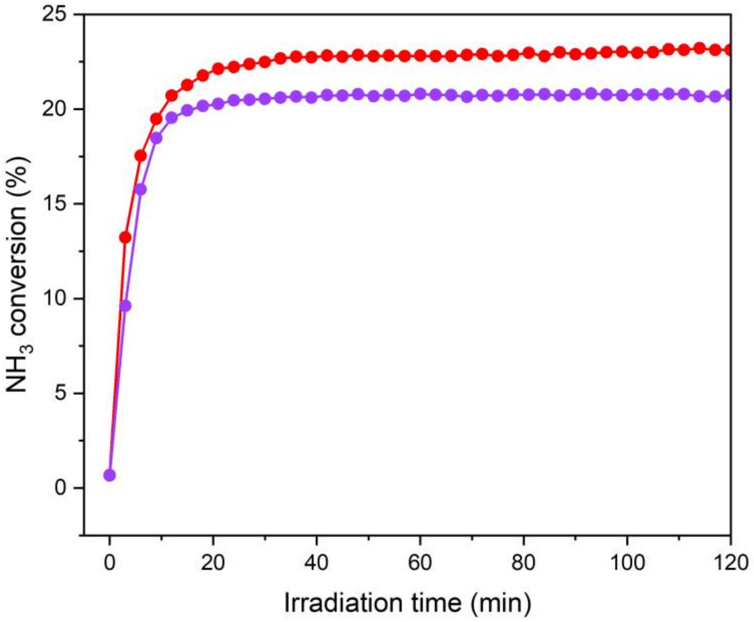

Figure S14. Conversion profiles for the photo-thermal ammonia decomposition reaction using KCC-1-NH_2-Ru@C-K (red) and SiO_2-NH_2-Ru@C-K (purple). Reaction conditions: 3.5 W cm^{-2}.

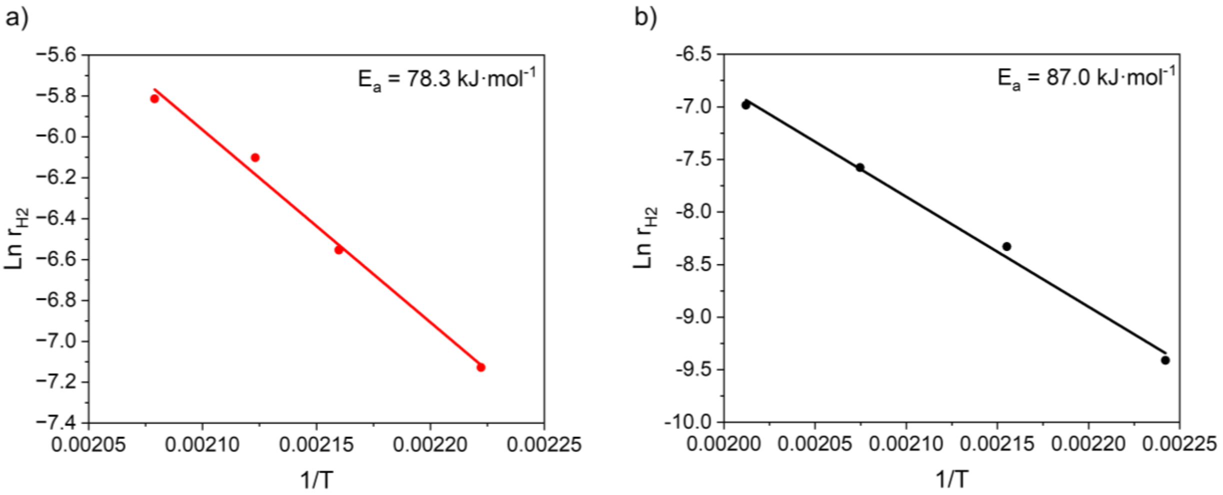

Figure S15. Arrhenius plots for NH_3 production rate under light (red) and dark (black) conditions over KCC-1-NH_2-Ru@C-K.

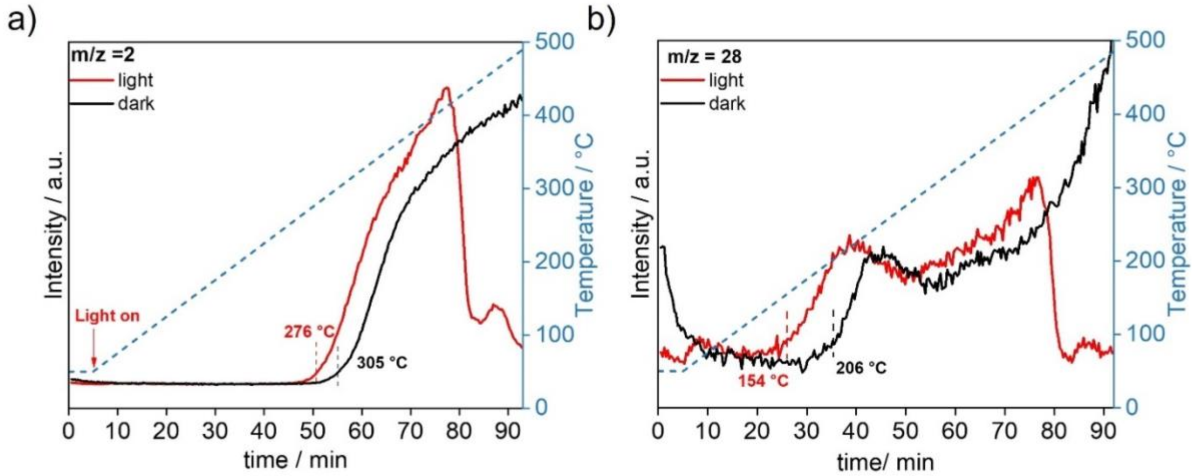

Figure S16. NH_3-TPD measurements on KCC-1-NH_2-Ru@C-K sample monitoring the formation of a) H_2 (m/z = 2) and b) N_2 (m/z = 28) under dark (black) and light (red) conditions using mass spectrometry.

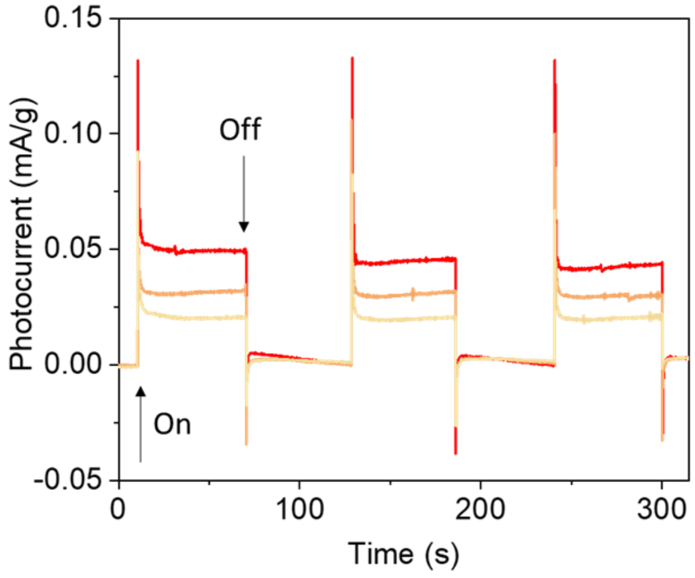

Figure S17. Transient photocurrent response under Xe lamp illumination (λ>420 nm) of KCC-1-NH_2-Ru@C-K under 400 (red), 300 (light orange) and 200 (light yellow) mW·cm^{-2} at -0.3 V vs Ag/AgCl.

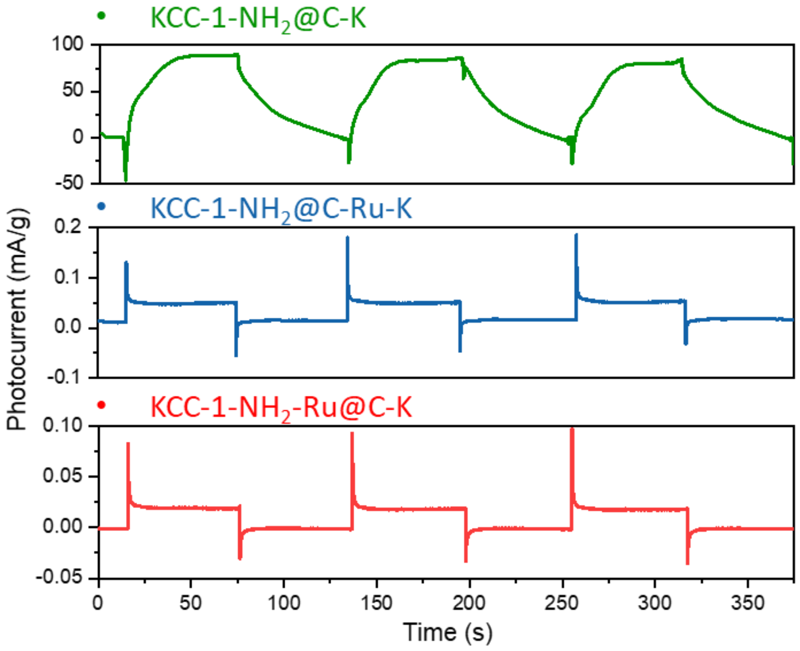

Figure S18. Transient photocurrent response under Xe lamp illumination (λ>420 nm) of KCC-1-NH_2-Ru@C-K under 400 (red), 300 (orange) and 200 (light yellow) mW·cm^{-2} at -0.3 V vs Ag/AgCl.

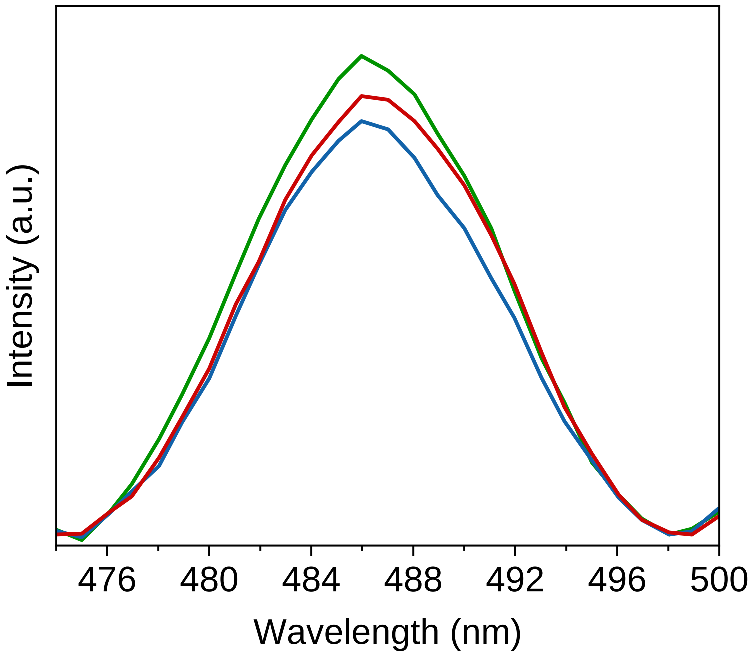

Figure S19. Steady-state photoluminescence (PL) spectra of KCC-1-NH_2-@C-K (green), KCC-1-NH_2@C-Ru-K (blue) and KCC-1-NH_2-Ru@C-K (red).

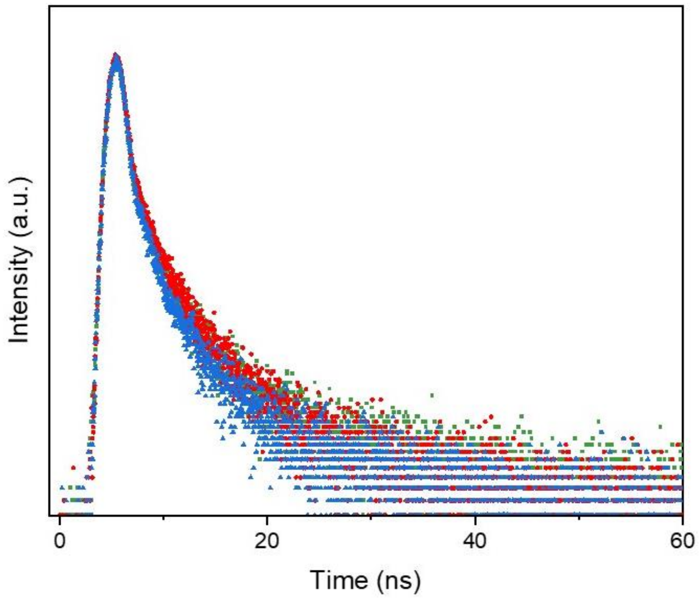

Figure S20. Time-resolved photoluminescence (TRPL) spectra of KCC-1-NH_2-@C-K (green), KCC-1-NH_2@C-Ru-K (blue) and KCC-1-NH_2-Ru@C-K (red).

Table S3. Fitted parameters of time-resolved PL spectra.

Sample	Component	Lifetime (ns)	Intensity (%)	Decay Lifetime (ns)
KCC-1-NH_2-@C-K	τ_1	5.28	53.8	4.77
	τ_2	0.79	46.2	
KCC-1-NH_2@C-Ru-K	τ_1	8.46	36.8	7.20
	τ_2	1.00	63.2	
KCC-1-NH_2-Ru@C-K	τ_1	9.59	41.2	8.31
	τ_2	1.22	63.2	

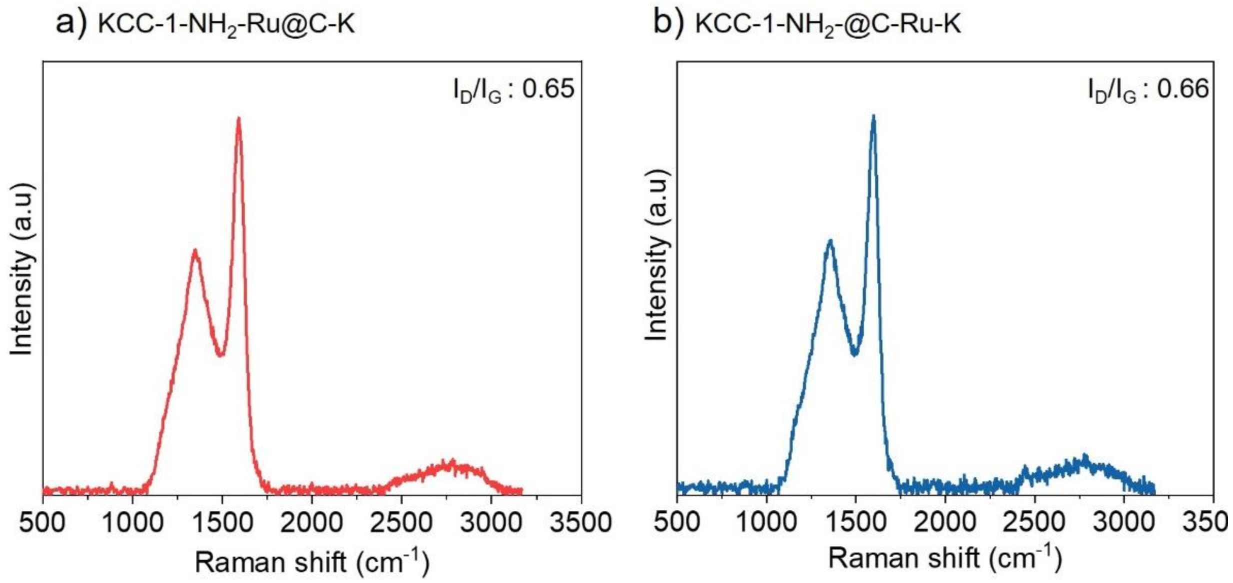

Figure S21. Raman Spectra of a) KCC-1-NH_2-Ru@C-K and b) KCC-1-NH_2@C-Ru-K.

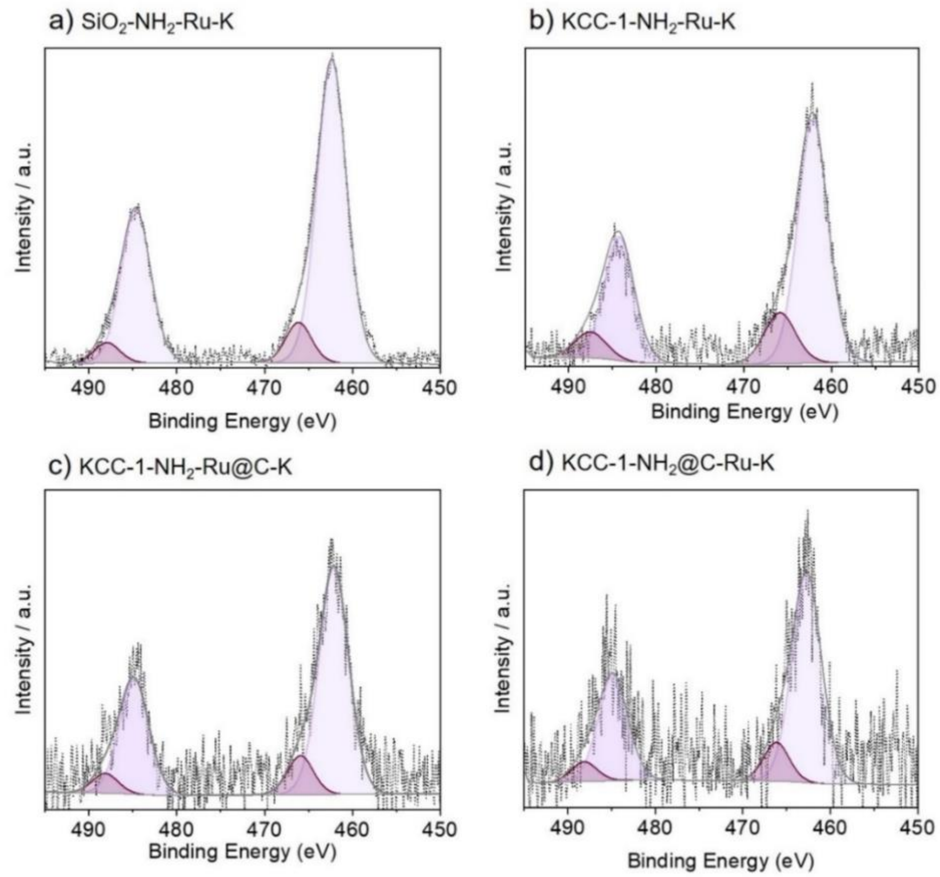

Figure S22. Ru 3p core-level XPS spectra of a) SiO_2-NH_2-Ru-K; b) KCC-1-NH_2-Ru-K, c) KCC-1-NH_2-Ru@C-K and d) KCC-1-NH_2@C-Ru-K.

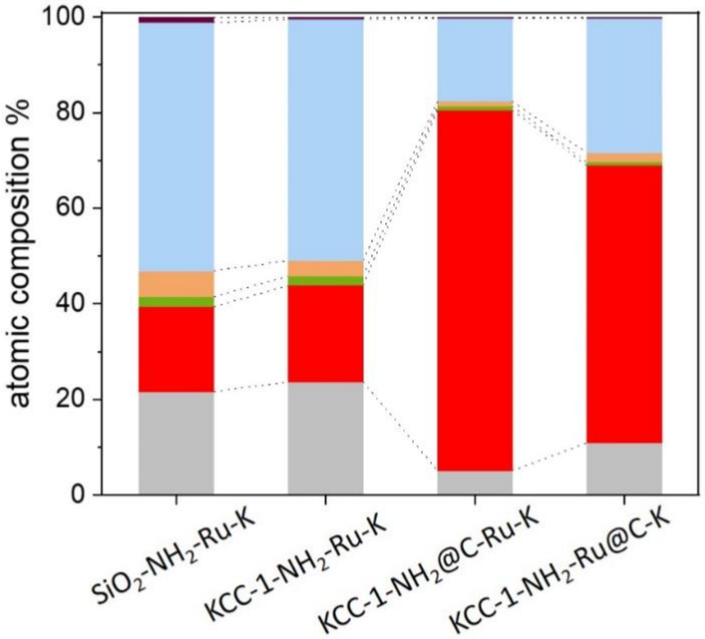

Figure S23. Elemental composition from XPS spectra of Ruthenium (maroon), Oxygen (light blue), Potassium (coral), Nitrogen (light green), Carbon (red), and Silicon (grey).

Table S4. Elemental composition from XPS spectra.

Sample	%	Si	C	N	K	O	Ru
**SiO_2-NH_2-Ru-K**		21.62	17.79	2.11	5.43	51.94	1.13
**KCC-1-NH_2-Ru-K**		23.54	20.10	1.92	3.23	50.23	0.45
**KCC-1-NH_2@Ru-K**		5.07	75.02	0.86	0.97	17.35	0.17
**KCC-1-NH_2-Ru@C-K**		10.80	57.76	0.68	1.94	27.98	0.25

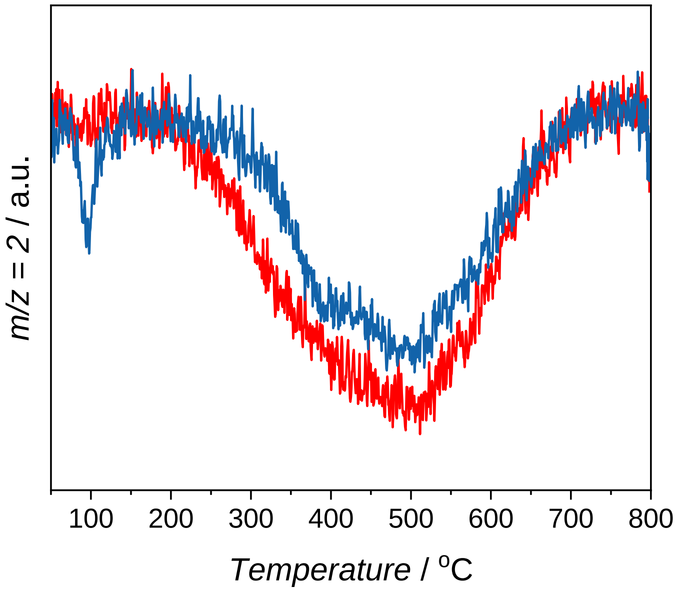

Figure S24. H_2-TPR profiles of KCC-1-NH_2-Ru@C-K (red) and KCC-1-NH_2@C-Ru-K (blue).

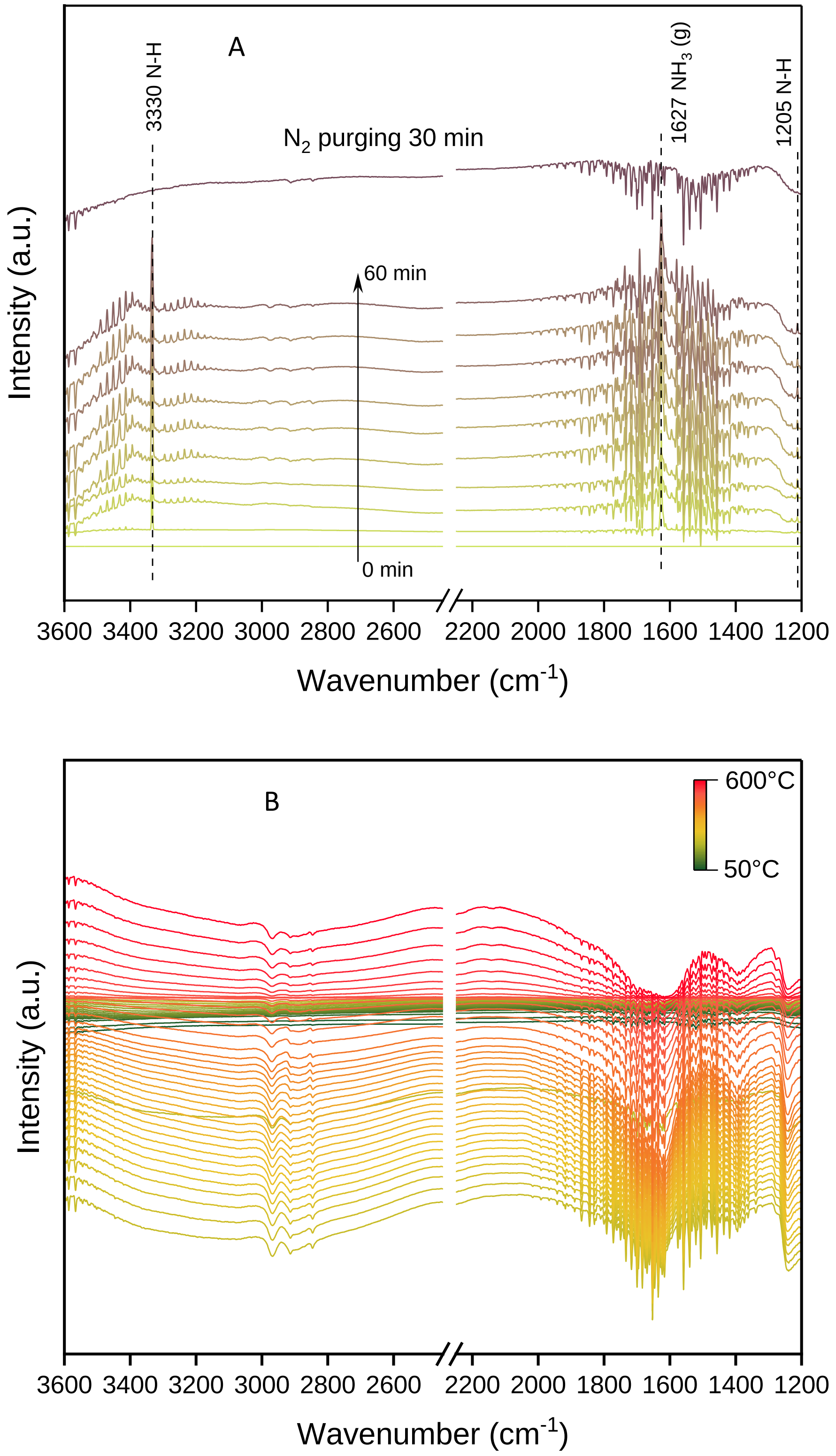

Figure S25. DRIFT experiments. In the panel a) we can see the adsorption the spectra of the KCC-1-NH_2-Ru@C-K while is contacted with a flow of 5% NH_3, the the sample is fluxed with N2. b) Panel b) shows the infrared spectra after being fluxed with N2 at different temperatures

1. Supporting tables

Table S5. NH_3 decomposition using Ru-based catalyst in thermal conditions.

Catalyst	Ru (wt. %)	WHSV ($mL·g^{-1}·h^{-1}$)	T (°C)	Conversion (%)	H_2 formation rate ($mmol\ g_{Ru}^{-1}\ h^{-1}$)	Ref.
**KCC-1-NH$_2$-Ru@C-K**	1.65	20000	300	23.0	17300	This work
**Ru/SiO$_2$**	10	30000	450	34.5	6840	[1]
**Ru/MCM-41**	5	30000	450	42.4	17040	[2]
**Ru/AC**	4.8	30000	450	28.7	12000	[3]
**Ru/CNFs**	3.2	6500	500	99.0	12360	[4]
**Ru/TiO$_2$**	4.8	18000	350	1.0	-	[5]
**Ru/Al$_2$O$_3$**	5	150000	500	11.0	21600	[6]
**Ru/MgO**	4.8	60000	400	22.0	18000	[7]

Table S6. Photothermal CO_2 hydrogenation using Ru-based catalyst.

Catalyst	Ru (wt.)	H_2 : CO_2	Reactor	WHSV ($mL·g^{-1}·h^{-1}$)	Light source	P (bar)	T (°C)	Productivity ($mmol·g^{-1}·h^{-1}$)	Selectivity (%)	Ref.
**KCC-1-NH$_2$-Ru@C-K**	1.65	3:1	flow	55418	300 W Xe lamp; 4.0 $W·cm^{-2}$	1	300	14000 $mmol·g_{Ru}^{-1}·h^{-1}$ (CO_2)	90 (CO)	This work
**2Ru/In$_2$O$_3$**	2	3:1	flow	120000	300 W Xe lamp; 1.41 $W·cm^{-2}$	1	~200	0.28 (CH_4)	18 .0	[8]
**Ru/TiO$_x$**	1	4:1	batch	-	300 W Xe lamp; 1 $W·cm^{-2}$	1	259.3	15.8 (CH_4)	99.9	[9]
**Ru/Mg(OH)$_2$**	8.3	1:1	batch	-	300 W Xe lamp; 18 suns	1	-	251 $mmol·g_{Ru}^{-1}·h^{-1}$ (CO)	46.0	[10]
**Ru$_{0.88}$Co$_{0.12}$/TiO$_2$**	2.57	4:1	flow	24000	300 W Xe lamp; 1.9 $W·cm^{-2}$	1	250	191 (CH_4)	94.6	[11]
**Ru/MnCo$_2$O$_4$**	1.3	4:1	flow	24000	300 W Xe lamp 1.25 $W·cm^{-2}$	1	230	66.3 (CH_4)	96.0	[12]

**Ru/MnO$_x$**	7.3	4:1	batch	-	300 W Xe lamp 2.5 W·cm^{-2}	10	200	166.7 (CH_4)	99.5	[13]
**Ru/Mg-CeO$_2$**	0.5	4:1	flow	200000	300 W Xe lamp 2.9 W·cm^{-2}	1	300	140 (CH_4)	70.0	[14]
**8 % Ru/SiO$_2$**	0.8	6:1	flow	309000	300 W Xe lamp 0.063 W·cm^{-2}	1	300	55.44 (CH_4)	99.0	[15]
**Ru-Al$_2$O$_{3-x}$-L**	0.7	4:1	batch	-	300 W Xe lamp 2.27 W·cm^{-2}	1	236	0.84 (CH_4)	99 .0	[16]
**Ru-Al$_2$O$_3$**	2.4	4:1	batch	-	300 W Xe lamp	1	375	95.75 % CO_2 conversion	99.2	[17]
**Ru/i-Si-o**	-	4:1	batch	-	300 W Xe lamp	-	~150	2.8 (CH_4)	-	[18]

Table S7. CO_2 hydrogenation using Ru-based catalyst in thermal conditions.

Catalyst	Ru (wt.)	$H_2 : CO_2$	WHSV (mL·g^{-1}·h^{-1})	P (bar)	T (°C)	Productivity	Selectivity	Ref.
**Ru@C-EDTA-20**	20	3:1	29880	1	200	13.8 μmol·g^{-1}·s^{-1} (CH_4)	100 %	[19]
**Ru/TiO$_2$-P25**	2.6	4:1	5760	20	300	2.6 μmol·g^{-1}·s^{-1} (CH_4)	100 %	[20]
**Ru/In$_2$O$_3$**	1	4:1	21000	50	300	0.57 $g_{methanol}$·g_{cat}^{-1}·h^{-1}	69.7 %	[21]
**Ru/a-TiO$_2$**	1	4:1	24000	1	300	5.2 % CO_2 conversion	>99 % (CO)	[22]
**Ru/r-TiO$_2$**	1	4:1	24000	1	300	5.7 % CO_2 conversion	96.3 % (CH_4)	[22]
**Ru (SAC)/FeO$_x$**	0.01	1:1	1200	1	360	14.5 % CO_2 Conversion	100 % (CO)	[23]
**Ru (NP)/FeO$_x$**	2.5	1:1	1200	1	360	15.6 % CO_2 Conversion	59 % (CH_4); 41 % (CO)	[23]

4. References

[1] Choudhary, T. V., Sivadinarayana, C., & Goodman, D. W. Catalysis Letters, **2001,** *72*, 197-201.

[2] Li, X. K., Ji, W. J., Zhao, J., Wang, S. J., & Au, C. T. *Journal of Catalysis*, **2005**, *236*, 181-189.

[3] Yin, S. F., Xu, B. Q., Zhu, W. X., Ng, C. F., Zhou, X. P., & Au, C. T. Catalysis Today, **2004**, *93,* 27-38.

[4] Xuezhi, D., Jinghong, Z., Gang, Q. I. A. N., Ping, L. I., Xinggui, Z. H. O. U., & De, C. H. E. N. Chinese Journal of Catalysis, **2010**, *31*, 979-986.

[5] Huang, C., Yu, Y., Yang, J., Yan, Y., Wang, D., Hu, F., & Feng, G. Applied Surface Science, **2019***, 476*, 928-936.

[6] Yin, S. F., Zhang, Q. H., Xu, B. Q., Zhu, W. X., Ng, C. F., & Au, C. T. Journal of Catalysis, ***2004***, *224*, 384-396.

[7] Yin, S. F., Xu, B. Q., Wang, S. J., Ng, C. F., & Au, C. T.. Catalysis Letters, ***2004***, 96, 113-116.

[8] B. Deng, H. Song, Q. Wang, J. Hong, S. Song, Y. Zhang, K. Peng, H. Zhang, T. Kako, J. Ye, *Applied Catalysis B: Environmental* **2023**, *327*, 122471.

[9] T. Dong, X. Liu, Z. Tang, H. Yuan, D. Jiang, Y. Wang, Z. Liu, X. Zhang, S. Huang, H. Liu, *Applied Catalysis B: Environmental* **2023**, *326*, 122176.

[10] N. Kong, B. Han, Z. Li, Y. Fang, K. Feng, Z. Wu, S. Wang, A.-B. Xu, Y. Yu, C. Li, *ACS Applied Nano Materials* **2020**, *3*, 3028-3033.

[11] Y. Tang, H. Wang, C. Guo, Z. Yang, T. Zhao, J. Liu, Y. Jiang, W. Wang, Q. Zhang, D. Wu, *ACS nano* **2024**.

[12] C. Guo, Y. Tang, Z. Yang, T. Zhao, J. Liu, Y. Zhao, F. Wang, *ACS nano* **2023**, *17*, 23761-23771.

[13] J. Zhai, Z. Xia, B. Zhou, H. Wu, T. Xue, X. Chen, J. Jiao, S. Jia, M. He, B. Han, *Nature Communications* **2024**, *15*, 1109.

[14] Z.-Y. Zhang, T. Li, X.-L. Sun, D.-C. Luo, J.-L. Yao, G.-D. Yang, T. Xie, *Journal of Catalysis* **2024**, *430*, 115303.

[15] C. Kim, S. Hyeon, J. Lee, W. D. Kim, D. C. Lee, J. Kim, H. Lee, *Nature communications* **2018**, *9*, 3027.

[16] X. Liu, C. Xing, F. Yang, Z. Liu, Y. Wang, T. Dong, L. Zhao, H. Liu, W. Zhou, *Advanced Energy Materials* **2022**, *12*, 2201009.

[17] X. Meng, T. Wang, L. Liu, S. Ouyang, P. Li, H. Hu, T. Kako, H. Iwai, A. Tanaka, J. Ye, *Angewandte Chemie International Edition* **2014**, *53*, 11478-11482.

[18] P. G. O’Brien, K. K. Ghuman, F. M. Ali, A. Sandhel, T. E. Wood, J. Y. Loh, J. Jia, D. Perovic, C. V. Singh, N. P. Kherani, *Energy & Environmental Science* **2018**, *11*, 3443-3451.

[19] J. Cored, A. García-Ortiz, S. Iborra, M. J. Climent, L. Liu, C.-H. Chuang, T.-S. Chan, C. Escudero, P. Concepción, A. Corma, *Journal of the American Chemical Society* **2019**, *141*, 19304-19311.

[20] A. Kim, C. Sanchez, G. Patriarche, O. Ersen, S. Moldovan, A. Wisnet, C. Sassoye, D. P. Debecker, *Catalysis Science & Technology* **2016**, *6*, 8117-8128.

[21] Q. Wu, C. Shen, N. Rui, K. Sun, C.-j. Liu, *Journal of CO2 Utilization* **2021**, *53*, 101720.

[22] X. Li, J. Lin, L. Li, Y. Huang, X. Pan, S. E. Collins, Y. Ren, Y. Su, L. Kang, X. Liu, *Angewandte Chemie International Edition* **2020**, *59*, 19983-19989.

[23] D. Zhang, J. Luo, J. Wang, X. Xiao, Y. Liu, W. Qi, D. S. Su, W. Chu, *Chinese Journal of Catalysis* **2018**, *39*, 157-166.
